# Design of Bio-Conjugated Hydrogels for Regenerative Medicine Applications: From Polymer Scaffold to Biomolecule Choice

**DOI:** 10.3390/molecules25184090

**Published:** 2020-09-07

**Authors:** Vittoria Chimisso, Miguel Angel Aleman Garcia, Saziye Yorulmaz Avsar, Ionel Adrian Dinu, Cornelia G. Palivan

**Affiliations:** Department of Chemistry, University of Basel, Mattenstrasse 24a, BPR-1096, 4058 Basel, Switzerland; vittoria.chimisso@unibas.ch (V.C.); miguelangel.alemangarcia@unibas.ch (M.A.A.G.); saziye.yorulmazavsar@unibas.ch (S.Y.A.); adrian.dinu@unibas.ch (I.A.D.)

**Keywords:** bio-conjugated hydrogels, biomolecule, polymer scaffold, regenerative medicine

## Abstract

Bio-conjugated hydrogels merge the functionality of a synthetic network with the activity of a biomolecule, becoming thus an interesting class of materials for a variety of biomedical applications. This combination allows the fine tuning of their functionality and activity, whilst retaining biocompatibility, responsivity and displaying tunable chemical and mechanical properties. A complex scenario of molecular factors and conditions have to be taken into account to ensure the correct functionality of the bio-hydrogel as a scaffold or a delivery system, including the polymer backbone and biomolecule choice, polymerization conditions, architecture and biocompatibility. In this review, we present these key factors and conditions that have to match together to ensure the correct functionality of the bio-conjugated hydrogel. We then present recent examples of bio-conjugated hydrogel systems paving the way for regenerative medicine applications.

## 1. Introduction

Regenerative medicine aims to restore damaged tissue by combining aspects of biomedical-, tissue- and genetic engineering, chemistry, material science and drug delivery. In order to achieve this, the cells need to be stimulated to grow and differentiate correctly by molecular signals that are generally growth factors (GFs), small drugs, proteins and nucleotide sequences [1,2,3]. Since the differentiation requires molecular cues, these need to be delivered to the cell in a sustained and continued way, whilst supporting cell growth [4].

In the past decades, one of the most significant challenges faced in regenerative medicine, tissue engineering and in vivo biomedical devices for tissue regeneration was related to the inability to mimic the mechanical properties and dynamic structure of the soft tissue that needed to be repaired or constructed [5]. Thus, research has been focusing on developing soft materials that: (i) are prone to adapt and resist physiological conditions, satisfying strict requirements for biocompatibility, softness, resistance to mechanical stress and general chemical versatility, (ii) provide the conditions for cell growth, differentiation and proliferation and (iii) degrade naturally or shortly after the healing of the damaged tissue [6,7]. These requirements can be fulfilled by hydrogels, which are cross-linked colloidal networks fully swollen in its entire volume by water if they are based on appropriate chemical nature of their building blocks [8]. Because of their high-water content and their flexible yet strong structure, the mechanical properties of a hydrogel resemble the consistency of soft tissue and hence can adapt to it, enabling their nowadays widespread use in commercial applications that range from contact lenses to hygiene products [9].

There is an almost never-ending list of examples of hydrogels, which have been designed and proposed as candidates for regenerative medicine, but their high cost or complexity can sometimes leave them behind in real life applications [10,11,12,13]. Commercially available hydrogels for tissue engineering are scaffolds, such as Mebiol^®^ gel and Corning ^®^ PuraMatrix™ peptide hydrogel, which are designed for cell growth and differentiation, and are used as external in vitro devices. Besides cell scaffolding, hydrogels are also employed in external wound dressing, and are composed of highly hydrophilic synthetic polymers frequently in combination with polysaccharides such as cellulose, alginates and hyaluronic acid [14]. Although their use in wound dressing is quite popular in regenerative medicine, the high production costs for more specific and finely designed hydrogels hinders their large-scale use in internal tissue engineering and drug delivery; thus, they have not found their way into the market yet [9]. Nevertheless, the examples on the market generally rely upon one synthetic or natural polymer, and not go any further in the use of natural biomolecules to widen their functionality [9]. Generally, a hydrogel network can be designed with any polymer, which has hydrophilic moieties, so that it is able to entrap water within its network [15]. By choosing the polymer, such to change its physical properties in function of an external stimulus, the hydrogel can respond to that stimulus by changing its mechanical properties and swelling behavior [16]. There is a large variety of hydrogels that are responsive towards light [17], pH [18], temperature changes [19], electrochemical potential [20], metal ions [21] and magnetic fields [22], as well as a combination of them [23,24]. These hydrogels are most commonly used in regenerative medicine due to their high biocompatibility, and tunable porosity, as well as for selective binding and protein purification, since the polymeric chains can be designed to have functional sites that specifically bind impurities or metals without impacting the stability of a protein [3]. What also distinguishes hydrogels from other materials is their extraordinary high-water content, which promotes the bioavailability of small molecules [25]. Hydrogels are also commonly used as drug delivery systems that can deliver their cargo over time or through a burst, depending on the target application [26]. It follows that this ability can be exploited to release a molecular signal overtime and induce cell differentiation and growth.

Polymers alone however are frequently inert, and normally function more as a scaffold or a carrier, as in the case of most commercial hydrogels, rather than an active material whereas pure biomolecule based hydrogels (e.g., peptides or proteins) can be toxic and trigger immune responses, leading to their degradation unpredicted and uncontrolled manners and lose their bioactivity [3,27,28]. Besides this, biomolecules are expensive to produce in large amounts and their resulting hydrogels are not only very costly but also mechanically weak [29]. In order to overcome the drawbacks of single component hydrogels, biomolecules and polymers have been combined to prepare bio-conjugated hydrogels [30]. These hydrogels carry both the properties of the polymers they are made of (e.g., hydrophilicity, inertness, or stimuli responsiveness), and the functional properties of the biomolecules (e.g., therapeutic, reversible sensing-actuation, cell binding, cell penetrating and adhesiveness) [31]. Besides the enhanced range of functionality that derives from tethering biomolecules to the polymer network, the cross-linked matrix of the hydrogel also provides spatial confinement, anchoring and overall protection to the biomolecule. Biomolecules serving to promote tissue regeneration are known as growth factors (GFs), and include proteins, DNA plasmids and non-coding RNA sequences. As the delivery of GF is still controversial from the ethical point of view, GFs are applied to nude mouse models that are frequently not sufficient to allow human trials [32]. Additionally, GFs have been also used as crosslinkers, contributing to both hydrogel bioactivity and mechanical properties whilst increasing biocompatibility. As we are focusing here on GFs, we will mention briefly other biomolecules, including carbohydrates, due to their high compatibility and low rejection rates, while for larger classes of molecules, including aptamers, lipids and steroidal hormones, we propose the reader excellent reviews in the field [33,34,35].

**Scheme 1:**
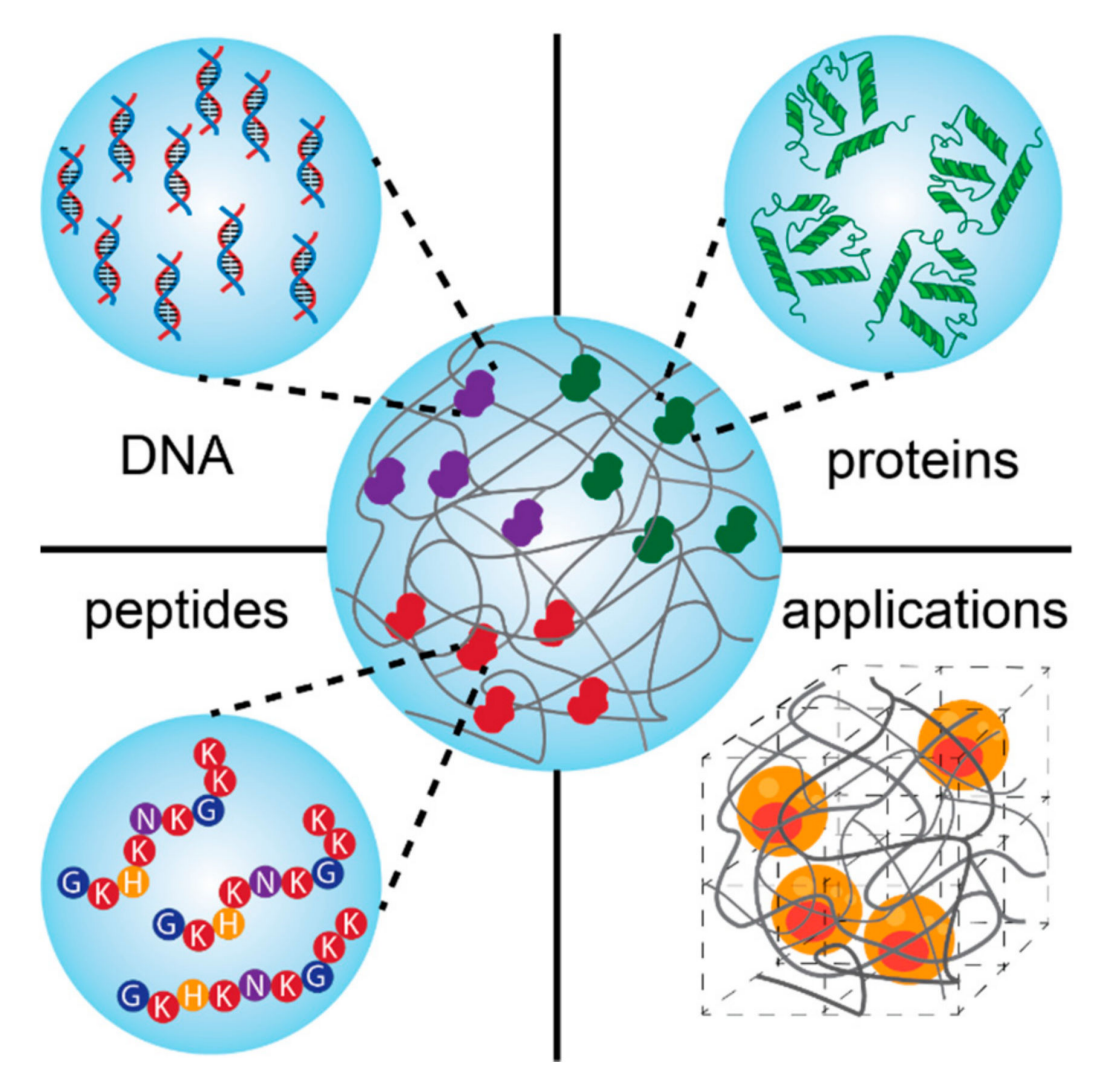
Schematic presentation of bio-conjugated hydrogels based on the combination of biomolecules (DNA, proteins, and peptides) and different polymers for regenerative medicine.

Here, we aim to give an overview about bio-conjugated hydrogels in which synthetic polymers and copolymers are combined with cell-active biomolecules with emphasis on their match as an important step to serve for successful application in regenerative medicine (Scheme 1). While there are a significant number of reviews on polymers and biomolecules used in regenerative medicine, a general overview on how to choose the polymer and the biomolecule according to the final use and the compatibility between these two classes of materials is still missing. With the goal to illustrate the molecular factors and conditions for an appropriate combination of polymers and biomolecules supporting the design of efficient bio-conjugated hydrogels, we present recent examples, and indicate the pathway that has to be taken in order to design and obtain these materials. We start with design criteria of a hydrogel for biomedical applications, followed by production of an appropriate hydrogel of interest because the design and synthesis of a bio-conjugated hydrogel poses a series of challenges when it comes to the combination of the synthetic systems with the biomolecule. Therefore, the way the hydrogel is coupled with the biomolecule, without the loss of the properties of any of the parts involved are explained here. Finally, recent examples of DNA, protein, and peptides-conjugated hydrogels are illustrated here to present how to design a bio-conjugated hydrogel for regenerative medicine and other biomedical applications, and which are their advantages and limitations.

## 2. General Factors Influencing The Design of Hydrogels for Regenerative Medicine

Each hydrogel is characterized by a series of physical and chemical properties that need to be specifically addressed when designing a tool for regenerative medicine. Features such as biocompatibility, gelling, degradation, porosity and intrinsic chemical properties, all play a role in the successful execution of the aim of the hydrogel [36]. For the choice of the polymer backbone, we redirect the reader to these outstanding reviews, which carefully and thoroughly cover the topic [36,37].

### 2.1. Biocompatibility

Any material, whether it finds in vivo or in vitro application on a biological system, requires high degrees of biocompatibility, in order to minimize cell or tissue damage. For instance, hydrogels for tissue scaffolding must be recognized by the body as not invasive, to minimize the general inflammation process and avoid damage to cells, surrounding tissue and fibrous encapsulation [38]. Natural polymers such as collagen or hyaluronic acid generally meet high standards of biocompatibility and are widely used in formulation and biomedical applications, yet lack of the freedom in tuning their chemistry and physical properties that synthetic polymers give [39]. Most synthetic hydrogels are considered potentially biocompatible if their chains are hydrophilic enough to host very high-water contents [36,40]. In vitro non-toxicity normally just relies on chemically nontoxic molecules and inert materials on which cells can proliferate without undergoing environmental stress. On the other hand, in vivo compatibility not only requires cell adhesion and proliferation but also requires hydrogel inertness towards the cell or tissue overtime, involves the recognition of the material as non-invasive, and demands no inflammatory response from the body. The fast reactivity of acrylates, methacrylates and acrylamides and vinyl monomers in both “free radical” and “living” polymerization normally ensures high yields in short times. However, these monomers also show high toxicity in soft tissue, and the difficult removal of these from the network can frequently hamper their use for in vivo applications [41]. For this reason, careful removal of all the monomer has to be performed before the implantation, either internal for tissue scaffolding or external as wound dressing device. An alternative to purification prior implantation is instead to reduce the lifetime of the monomers within the tissue before being polymerized. Some polymers can be also problematic upon degradation: in the case of acrylamides such as poly(*N*-isopropylacrylamide) (PNIPAM), the hydrolysis of the side chain leads to the formation of small amine compounds which are cytotoxic [42]. For this reason, in the past years a great alternative to PNIPAM has been poly(*N*-vinylcaprolactam) (PVCL), which has comparable responsiveness to temperature. Regardless of the size of the hydrogel, both macro-, micro- and nano-gels have shown to be successfully employed in vivo. However, the higher complex chemical and biological environment that these hydrogels must face can lead to side reactions and interactions. For instance, the immune response towards the hydrogel must be minimized or repressed in order to avoid the damage to surrounding tissues and to promote the activity for which the hydrogel has been designed and implanted. In vitro tests generally involve cell viability, metabolic activity and cell proliferation, which indicate no toxicity in simple environments. Cytotoxicity however does not necessary imply biocompatibility, which requires immunogenicity, mutagenesis and proteomics test [43]. There is a fundamental difference in the application of polymers for in vivo or in vitro application. Specifically, some polymers like poly(ethylene glycol) (PEG), which are the milestone for in vitro cell culture due to their unique hydrophilicity and inert character, makes them the perfect candidates for cell culture and scaffolding. However, the same polymer seems to be less tolerated in vivo, since recent studies have identified the presence of anti-PEG antibodies, which clearly lead to the material rejection and inflammation of the surrounding tissue [44]. To further promote the biocompatibility of a polymer, frequently the device is decorated with biomolecules like heparin, which reduces thrombogenicity. [45] Further functionalization includes the use of albumin or RGD peptides which also promote cell adhesion and growth factors [45]. Moreover, by adjusting their porosity and micropatterning, the surface will increase their wettability leading to an overall higher biocompatibility level, as cells tend to recognize the material [38].

### 2.2. Gelling and Mechanical Properties

The gelling of a polymer is achieved by forming permanent or reversibly covalent cross-links between the polymeric chains or can arise by the formation of physical ones that induce a viscoelastic character to the system [46]. Covalent macro-gels are normally obtained via photo- or thermal-initiated polymerization and rely on classic hydrophilic cross-linkers [36]. Small hydrogels, in the size range of 10–100 μm, are generally formed by exploiting microfluidic or molding techniques, whilst even smaller microgels (10 nm to 1 μm) are obtained with precipitation or emulsion polymerization [47]. Generally, covalently-bound hydrogels are characterized by a predominantly elastic behavior, and can undergo axial deformation, sustain stress and mechanical friction and corrosion, whilst they respond badly to shear forces [48]. The Young’s modulus depends on the nature of the polymer and on the cross-linking density, as well as on the wt% of mass content of the hydrogel. Covalent hydrogels composed of a single network, are generally stiff and brittle: the fracture energies are low (about 10 J m^−2^) and so are the Young’s moduli [49,50]. Once the chains are broken and are subjected excessive strain, the deformation becomes irreversible, and ruptures and cracks appear. Cartilage implants require significantly higher fracture energies, and thus are based on double interpenetrating networks, since their fracture energies and Young’s moduli are magnitudes higher [48,51]. Preformed covalent hydrogels are generally used for external applications or inserted via invasive surgery, exemption made for microgels, which have a radius significantly smaller than the injection needle, and macroporous hydrogels, which benefit from the flexibility that arises from the high volume of pores [52,53]. When injected, macroporous hydrogels expel water from the pore cavity and regain the original shape, when extruded from the needle by taking up fluid from the surrounding environment [54]. However, they are problematic to adapt to the new environment as it presents an irregular shape that cannot be filled out completely. Moreover, the safety and in vivo efficacy of these materials has to be confirmed yet [55]. An alternative to them is to inject highly concentrated microgel solutions which, due to their soft and fuzzy structure, can be easily injected and deformed and have proven to prolong their blood circulation time. Since these hydrogels have a high surface area and are subjected to shear forces in the blood, the polymer needs to be stable enough to minimize erosion. For instance, covalently cross-linked PNIPAM resists well to erosion as it is not only covalently cross-linked, but undergoes further self-crosslinking by proton abstraction in the tertiary H of the main chain, while the more biocompatible PVCL is structurally not able to undergo similar self-crosslinking [56]. PVCL is thus more sensitive to shear and tends to decompose starting from the outer corona when subjected to low shear forces [57]. It has been recently demonstrated that even manual extrusion from a syringe leads to the cleavage of the outer corona in PVCL microgels, making them problematic for applications that require injections. On contrary, PNIPAM microgels subjected to low shear forces maintained their structural integrity overtime, with shear moduli ranging from 0.221 kPa to 0.015 kPa depending on the cross-linking density.

If the cross-linking derives from supramolecular interactions, such as hydrogen bonding, host-guest chemistry or metal ligand interaction, the class of hydrogels is known as supramolecular hydrogels [58,59]. Supramolecular hydrogels change their mechanical properties when subjected to shear and are thus injectable when they display shear-thinning properties [60]. The rather labile bonds can break more easily compared to their covalent counterparts and are therefore the sacrificial bonds that break when shear is applied, leading to the flow of the polymer hydrogel. Since the bonding and de-bonding of the cross-links is reversible, the hydrogel can regain its original mechanical properties once the strain is removed [59]. On the other hand, the shape is not retained, and the hydrogel assumes the new shape of the container in which it is molded, as if it was a liquid. The viscoelastic behavior plays a pivotal role when the hydrogel must be injected in a tissue cavity, since it can adapt to its irregular shape [3]. The injection of shear thinning self-healing hydrogels is preferred to the in-situ gelation of a polymer mixture, since it prevents the dispersion of the single polymer chains or monomers before gelation. Furthermore, countermeasures to the leakage and contamination have been taken by diametrically switching the approach in gelling kinetics [61]. The initial idea of in-situ gelling was to have a hydrogel that undergoes the stiffening transition extra fast when outside the needle. In recent years, a slow but steady gelling kinetics has proven successful, easier to handle and with reduced toxicity [62]. For example, if the covalent cross-linking derives from the reaction between a hydrazine and an aldehyde, exchanging the aldehyde for a ketone functionality leads to a reduction of the gelling kinetics [61]. This approach not only lowered enough the gelling to achieve injection, but also biocompatibility and transparency is dramatically enhanced. The cross-linking between the macromers starts already in the needle, yet it is slow enough to permit the loosely cross-linked polymer chains to be extruded through the syringe, adapt to the cavity and finish cross-linking without any major contamination of the surrounding tissue [63]. In recent years, self-healing materials based on supramolecular chemistry have offered fast reversibility in their mechanical properties and structure regeneration under mild conditions and have become an important research topic in materials design [64]. They display excellent mechanical properties, which derive from the reversible supramolecular network in the presence of secondary interactions that bind liquid-like building blocks into polymers in hydrated and non-hydrated states. Hence, non-covalent bonds are the most reliable to be applied as self-healing “smart” cross-linkers or hydrogel macromers. Several chemical approaches have been applied using a combination of non-covalent bonds, such as hydrogen bonds [65], π-π stacking [66], electrostatic interactions [67], metal-ligand interactions [68], hydrophobic interactions [69], and host-guest interactions [70].

### 2.3. Architecture and Shape

The architecture is a key factor influencing the hydrogel’s physical behavior, namely gelling, responsiveness towards stimuli, as well as mechanical properties. For instance, PNIPAM can form physical hydrogels above its lower critical solution temperature (LCST) by undergoing a coil to globule transition, as it becomes less hydrophilic in water. This decrease in hydrophilicity leads to the formation of hydrophobic domains, which can act as physical cross-linkers when the PNIPAM chains are above their critical gelation concentration (CGC). However, the resulting hydrogel displays extremely poor mechanical properties when subjected to shear or stress [71]. Since the LCST value for PNIPAM [72] (and PVCL) is 37 °C [73], which is also the average body temperature, this gelation method is applied in certain cases for in situ gelling when high elasticity and toughness are not required. Other polymers that present biomedical relevant LCST are PNIPMAM (38 °C) [74], poly(*N*,*N*-diethylacrylamide) (PDEAAM) (32–34 °C) [75] and poly(methyl vinyl ether) (PMVE) (37 °C) [76]. The mechanical properties of hydrogels can be endowed with temperature responsiveness once a cross-linker is introduced, and the Young’s modulus can be tuned as well by changing the cross-linking density [77]. Besides the cross-linking density and distribution, the molecular weight and flexibility of the network forming polymer chain must be considered carefully as the inter-chain entanglements and loops also contribute to the overall toughness or resistance of the hydrogel, in addition to the influence on the CGC [78]. Macroscopic hydrogels do not necessarily have to be composed by linear chains cross-linked by small fragments. Contrary, there is a plethora of examples of hydrogels that find wide applications in the biomedical field that are composed by macromers with higher complexity architectures [79]. Star polymers can be covalently or non-covalently linked one with each other to form a hydrogel matrix [80]. Even though the synthesis of the hydrogel precursor requires more effort that using a single monomer, the tendency of the arms of star polymers to align rather than to progress in random coil prevents the formation of too high entanglements and loops. The dispersity of macromers impacts the reproducibility of the mechanical characteristics of the gels. In fact, this can be observed well in hydrogels composed from hyperbranched polymers and dendrimers. Whilst hyperbranched polymers are highly disperse and the branching is random, dendrimers are highly defined structures that are almost monodisperse macromers after purification [81]. Different batches of macroscopic hydrogels can vary greatly when composed of hyperbranched polymers, whilst a hydrogel derived from a dendrimer of the same chemical composition will show reproducible and fixed characteristics [82]. Both architectures ensure a dynamic inhomogeneous structure of the overall hydrogel [83]. The varying density of the macromer throughout its structure reflects then the variation of local chain density of the macroscopic hydrogel. This inhomogeneity resembles the uneven structure of the extracellular matrix and allows the cells to proliferate better, also because of it dynamic continuously re-modelling. Moreover, the extremely high number of terminal ends also decreases the CGC [84]. Structural complexity can not only be achieved in 3D polymers, but also in 2D, where different blocks in the same polymer express significantly different physical properties. Block copolymers can self-assemble into nanostructures with local phase separation that can lead to organized cross-linking, leading to hydrogel formation [85]. The most popular block copolymers are composed of di- or triblock domains. Diblock copolymers based on a hydrophilic and a hydrophobic domain generally lead to the gelation of sample at higher wt% concentrations compared to the triblock counterparts [86]. This behavior is attributed mainly to the intrinsic ability of triblock copolymers to interact both inter- and intramolecularly, leading to an increase of interactions and thus drastically reducing the CGC [86].

For microgels, a variety of architectures has been explored and proposed for biomedical applications, mostly as candidates for drug delivery applications that involve the survival of the hydrogel in the blood stream, or in bone marrow regeneration [87,88]. Microgels can be isotropic or anisotropic in size, and can have a homopolymer, core-shell, core-shell-shell, hollow or Janus architecture [89]. All these provide the microsystem with tunable functionalities given the chemical properties of the different polymers. Compared to macroscopic hydrogels, microgels respond much faster to environmental changes, namely temperature, pH, light, and so on [47]. Their faster responsiveness is correlated with their architecture: the soft and fuzzy structure combined with their small dimension leads to almost instant changes in local polarity, solubility and size.

### 2.4. Porosity and Network Density

**Figure 1:**
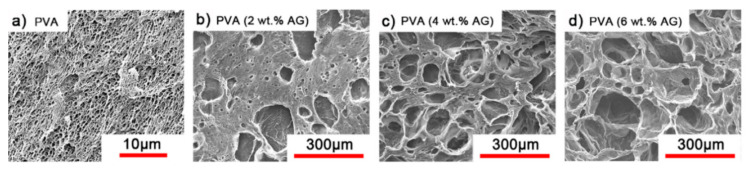
Scanning electron microscope (SEM) micrographs of dried (**a**) PVA hydrogel and (**b**–**d**) macroporous PVA hydrogels treated with (**b**) 2 wt% (**c**) 4 wt% and (**d**) 6 wt% of agarose as pore formation agent [93].

For a series of biomedical applications, that go from tissue regeneration to drug delivery, porosity is a parameter to regard, as it influences the diffusivity of a small molecule and the proliferation of cells within the network [90]. Each application requires a range of pore sizes that is between 5 and 100 nm, and increasing up to 400 μm. For a hydrogel to be applied in drug delivery, the width of its pores has to be designed as function of the size, specifically the hydrodynamic radius, of the drug or cargo that has to be delivered [26]. Generally, small molecules such as antibiotics move freely through the network. If the pores are larger than the cargo, the release of the cargo is dominated by diffusion [91]. By reducing the pore size to a 1 to 1 ratio with the cargo, the cargo does not diffuse freely through the network anymore, and its release is slowed down. This is mostly due to the longer path that the cargo has to take when is sterically hindered by the network chains. Moreover, by increasing the network density, some pores will be smaller, lengthening the path of the cargo. By reducing the size of the pores to a ratio smaller than one, the cargo is entrapped within the network. In this case, the main application of the hydrogels is the long-term storage of the cargo, which can be released by erosion. Tissue regeneration requires porosities that allow the cells to proliferate and diffuse through the network; thus, the pore sizes should typically be in the 100–300 μm range. These also need to promote the growth of blood vessels and general vascularization. A certain polymer network can be organized in different pore sizes according to the formation conditions. The final porosity strongly depends not only polymer content and cross-linking mol% and density, but also on the formation temperature, solvent composition and solvent exchange [92]. Moreover, the introduction of a pore-forming agent (i.e., agarose), as in the case of a PVA hydrogel, can induce the formation of macropores that can be used in cell scaffolding (Figure 1) [93].

Some applications also require the change in porosity either instantly or over prolonged periods. To address these two requirements, generally degradable and responsive polymers are used in the network design. By introducing a stimuli-responsive polymer in the network, the subsequent shrinking or swelling due to an external stimulus drastically changes the pore size and the release kinetics [94,95]. For instance, thermo- and pH responsive microgels composed of VCL and itaconic acid (IA) would release cytochrome c (cyt-c) in different kinetics depending on the external conditions. An increase in temperature above the volume phase transition temperature (VPTT) of the chain network or a reduction of the pH below the pKa would lead to an immediate collapse of the pore size, and a burst release of the protein [95].

### 2.5. Degradability

How long the polymer will retain its structural integrity overtime and its resistance towards mechanical stress, erosion and enzymatic and oxidative stress, have to be regarded in function of the application desired for the device composed of the polymeric material. In the cases of external devices, generally a long-lasting material composed of aliphatic main chains and not hydrolysable cross-links is used. The chemical stability that arises from the covalent single bonds makes polymers made out of vinyl monomers the most popular ones. The main chain has to undergo a series of reversible conformation changes, and last over a high number of cycles. In this case, a covalent single or double network is preferred. When the polymer is used to build a scaffold, this should mimic as good as possible the behavior of the extracellular matrix (ECM), by either giving space to the tissue to grow overtime and gradually disappear from the implantation site or dynamical moving away to make space for the growing tissue. This is achieved by relying on the lability of certain bonds within the network. According on how the polymer network will break down into smaller segments, a polymer is degradable or resorbable [96]. If the monomers are biocompatible and do not produce any damage to the surrounding tissue, this is the preferred approach. However, the most popular biodegradable polymers PLA and PLGA lead to a decrease of the tissue pH value since they produce lactic and glycolic acid, inducing inflammation of the surrounding tissue [97]. Inflammation normally occurs as the hydrolysis of PGA into glycolic acid has been proven to activate the classical complement pathway, which is an immune response that involves antigen antibody systems. In fact, glycolic acid binds immunoglobulin far more than the polymer, hence inducing the inflammatory response due to what is commonly known as an allergic reaction [98]. To avoid or reduce the inflammatory response, decoration of the surfaces with heparin has been proven useful. Other polymers are biodegradable, but very few do not produce allergic response when they break down into their former monomers. An example for this class of polymers is ethylene-vinyl acetate copolymer, which provoked mild to no inflammation even after 3 or 4 weeks after implantation [99]. The alternative degradation method revolves around polymeric chains that break down into large chains, yet small enough to be filtered through the kidney system. The kinetics of degradation of a polymeric construct has to be tailored by carefully choosing the chemistry of the main chain and cross-links [100]. Degradation in longer chains involve the cleavage of labile bonds, that can be attacked either chemically (pH changes) or enzymatically (esterase to cleave ester bonds). The most common labile bonds are esters, digested either by low pH or an esterase, and disulfide bonds that can be cleaved by an addition of a reducing agent [101]. If the main chain is not degradable, it is necessary to introduce cleavable moieties, that breakdown overtime or ‘on demand’ by changing the physical conditions or adding an enzyme to digest specific bonds [102]. Temperature- and pH-responsive microgels were cross-linked with a disulfide cross-linker and could be degraded upon the addition of DDT. Degradation can also occur via the erosion of the network by spontaneous break down of the network chains from the surface when subjected to frequent stress. Erosion is mostly uncontrolled in materials that contain amide or ester bonds, which can be hydrolyzed under mild conditions. However, erosion is another way to deconstruct gradually the network from outside. This is achieved by exploiting supramolecular cross-linking [94]. The erosion rate can be fine-tuned by changing the host-guest interaction or by changing the amount of cross-linkers within the network.

## 3. Synthetic Criteria for Hydrogel Production

Carefully choosing the synthetic path to build a polymeric scaffold for biomolecules requires retaining and enhancing the properties of the whole material. Since most complex biomolecules are sensitive to changes in the external environment and easily undergo denaturation, the bio-conjugated hydrogels must be engineered to accommodate and protect these delicate payloads. Considering these key points, it will allow for both building polymeric scaffolds endowed with versatile properties and preserving the functionality of the entrapped/attached biomolecules.

### 3.1. Polymer Choice

**Figure 2:**
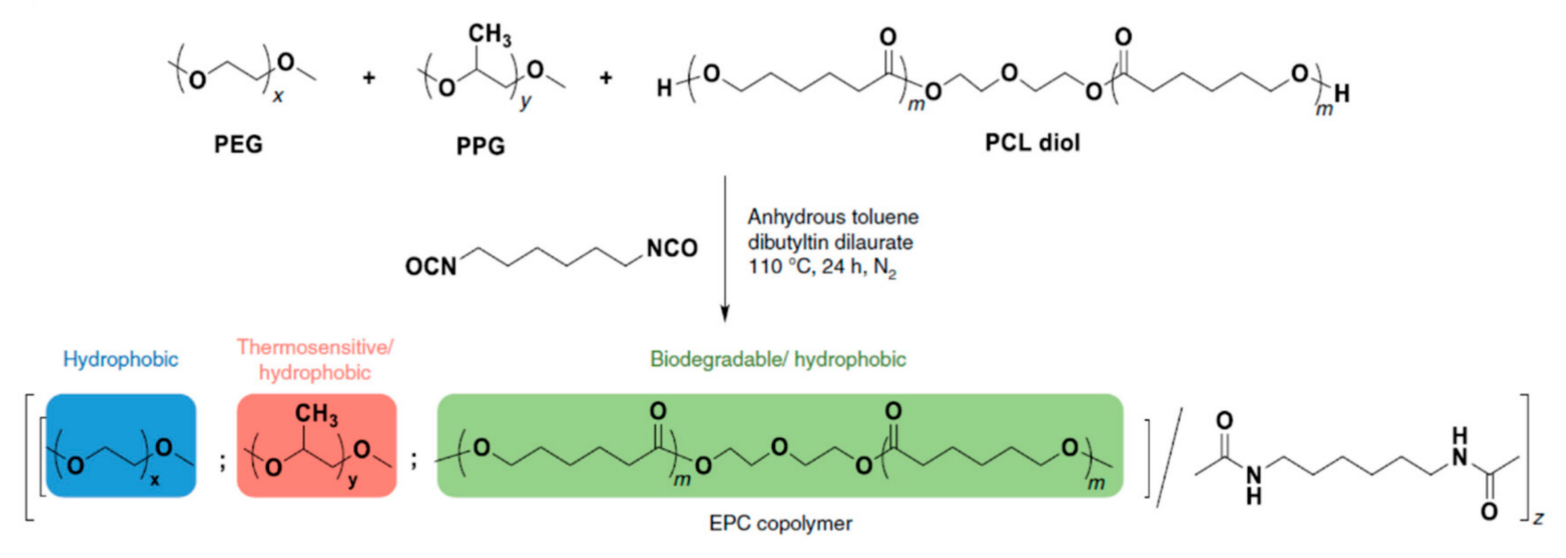
Schematic representation of the polymerization procedure for synthesis of a biocompatible and biodegradable EPC copolymer [119].

The polymer backbone defines the stiffness, elasticity and degradation of the polymer chains. Depending on their chemical nature, the resulting hydrogels will be biocompatible, biodegradable or intrinsically responsive to various stimuli (Table 1). Degradable polymers containing cleavable moieties within their backbone can reduce their molecular weight overtime, until they are shortened to units small enough to be exerted by the body. A prominent example of degradable synthetic polymer is PLA and its corresponding PGA, which are polyesters that undergo hydrolysis in mild acidic conditions or can be cleaved ‘on demand’ by an esterase [103]. Such polyesters are obtained via ring-opening polycondensation [104]. The two stereoisomers, D-lactic acid and L-lactic acid, influence both the hydrogel mechanical properties as well as its degradation rate [105]. Degradation of a hydrogel into the human body (T = 37 °C) is also dependent on the temperature in aerobic conditions, the polymer molecular weight and crystallinity [106,107]. Thus, the careful choice of a second comonomer enables the fine-tuning on how the polymer will degrade and how fast. Besides its low production costs and easy biodegradability, PLA also comes from renewable feedstock [108]. However, it lacks of any kind of responsivity towards external stimuli. Similarly, PCL also degrades with different rates according to its composition and external conditions, whilst remaining bioinert [109]. To enhance the bio-functionalization, synthetic polyesters from tailored monomers can have instead their chemical and mechanical responsiveness tuned through monomer chemical nature. For instance, polythioesters with a functional tertiary amine display facile functionalization and allow further biomolecule coupling [110]. Polyesters are flexible biodegradable and resorbable polymers, and come in hand for valves and tissue regeneration applications [111,112,113,114]. Instead, if the need of a bioinert, stronger and tougher polymer is required, polyurethanes (PUs) are the polymers of choice. PUs are obtained by polycondensation of polyols with *n*-functional isocyanates [115,116,117,118,119,120]. The hydrogels containing biodegradable PUs generally lack elasticity, due to the high crystallinity of the chains. Introducing a trifunctional triol to be reacted together with a bi-functional diol ensured the formation of a material with great elasticity that resembles the ECM [121]. Changing the ratios of isocyanate and triol allows to modify the microstructure and chemistry of the PU hydrogel. Moreover, by connecting together different segments based on hydrophilic PEG, thermosensitive poly(propylene glycol) (PPG) and the biodegradable and hydrophobic poly(ε-caprolactone) (PCL) via urethane bonds (Figure 2), a biocompatible thermogelling polymer (EPC) was engineered to provide an internal tamponade effect through surface tension and swelling counterforces for the repairment of retinal detachment [119]. During its biodegradation, the thermogel promotes the reformation over time of a vitreous-like body mimicking the biophysical properties of natural vitreous body.

If a specific response towards an environmental change is required, the use of stimuli-responsive polymers is necessary. For example, a stimuli-responsive monomer can be blended into the comonomer composition of an inert polymer donning a triggered responsive character to the resulting hydrogel. Clearly, there are ranges of comonomer compositions and ratios that have to be respected to obtain the given responsiveness [122]. Polymers that show temperature responsiveness, be it either an LCST or upper critical solution temperature (UCST) behavior, require a monomer loading of at least 70%. This has been observed for the most common temperature-responsive polymers, i.e., PNIPAM, poly(*N*-isopropylmethacrylamide) (PNIPMAM) and PVCL.

**Figure 3:**
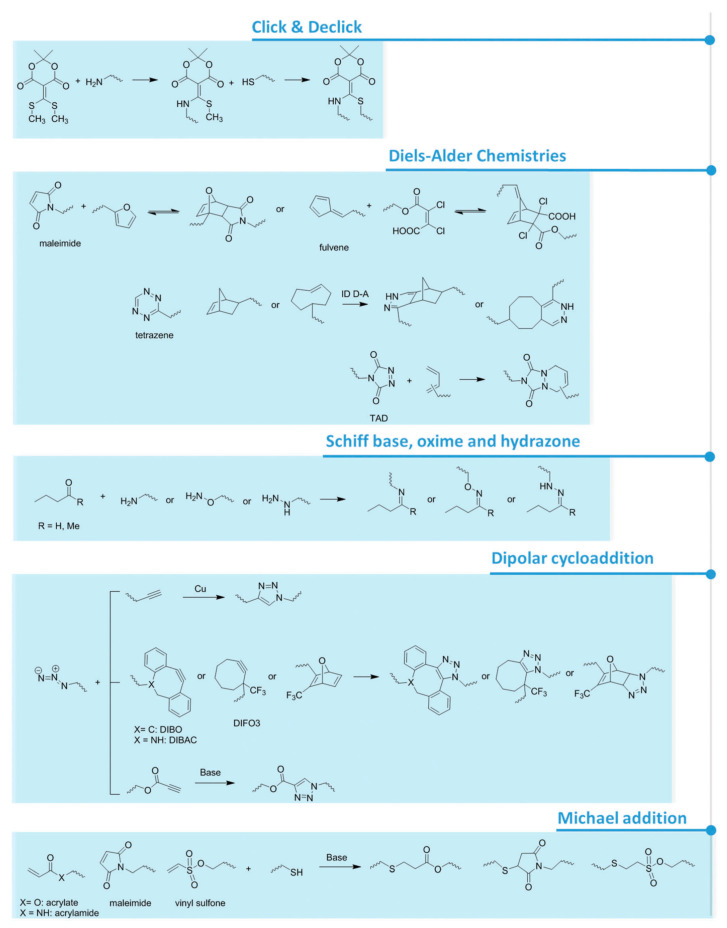
Schematics presenting several functional groups involved in hydrogel formation [128] and attachment of biomolecules via covalent bonding.

For what concerns pH responsiveness, lower loadings can be achieved whilst maintaining a responsiveness of the main chain. Already a 2 to 5% comonomer composition of acrylic acid (AAc) or methacrylic acid (MAAc) leads to swelling and deswelling of the overall gel [123]. Furthermore, a content of repeating units comprised of light-responsive moieties, as low as 1%, can change the chain polarity and thus the polymer behavior in solution [123]. Besides the most structurally simple polymers, like PEG, polymers that contain a functional side group come in hand for post-polymerization coupling of biomolecules or for the formation of chemical or physical cross-links within the hydrogel network. This approach involves the polymerization of monomers that bear a functional group that will not be attacked or degraded during the polymerization, but can then be modified and reacted afterwards [124,125]. Representative examples include PAAc and its analogues, polyamines and analogues, and poly(NHS) esters. Depending on the type of functional groups attached to the polymer backbone, the possible reactions used for hydrogel formation or biomolecule coupling involve active esters, anhydrides, isocyanates, epoxides, Michael addition reactions, cross-coupling moieties and click chemistry reactions (Figure 3) [52,101,124,126,127,128]. Besides, the functional side groups can also form physical cross-links by hydrogen bonding as in the case of ureidopyrimidone [129] or polyphenol-based patterns and crystallization [130]. Other approaches to prepare hydrogels involve host-guest supramolecular interactions, where one polymeric chain is generally decorated with the host molecule and another one with the guest(s). Relevant examples include hydrogels based on binary complexes of cyclodextrin and adamantine [131], and the ternary complexes of cucurbit[n]urils, which have been successfully implanted into the mice brain since they perfectly mimic the consistency and mechanical properties of neuronal tissue [132].

Clearly, most moieties that are able to react with functional groups belonging to biomolecules, such as an NHS group, have the main drawback to have scarce solubility in water or other biomolecule-friendly solvents. To overcome this problem, copolymerization with a water-soluble monomer generally allows 10 mol% loading of the functional groups within the polymeric backbone, increasing the solubility of the otherwise insoluble reactive moiety [133]. However, the synthetic networks that provide a base for biomolecules do not have to affect the biomolecule integrity or activity.

### 3.2. Polymerization Conditions

In the case of macroscopic hydrogels, free radical polymerization, which does not provide any sort of control on the chain dispersity, is still a strong tool to obtain freestanding gels with a statistical comonomer distribution [161]. From all polymerization techniques, this one, even though air-sensitive, is one of the most robust ones. When a precise control over the chain length dispersity and molecular weight of a polymer are required, ‘living’ polymerizations are rather advised [162]. However, the hydrogels prepared by ‘living’ polymerizations involving metal ions, e.g., ATRP and ROMP, need to be purified thoroughly [163]. Alternatively, metal-free “living” polymerizations such as RAFT, MADIX, anionic and cationic are rather recommended to ensure high molecular weights and narrow dispersity in the block length distribution [162,164,165].

During the polymerizations in which the biomolecules are co-polymerized/encapsulated within the hydrogel network, several key parameters, including temperature, solvent and pH, need to be fine-tuned to ensure both polymerization and preservation of the integrity and functionality of the biomolecule. For instance, conducting the polymerization at temperatures higher than 60 °C can lead to the denaturation of proteins and other biomolecules. To overcome this problem, light-induced radicals are exploited, which lowers the temperature required to carry out the polymerization, whereas for UV-sensitive biomolecules there is a wide variety of light-sensitive initiators that generate radicals at RT and longer wavelengths. Proteins or DNA are not only sensitive to temperature, but also towards pH. Polymerization that require extreme pH values to ensure the solubility of a monomer are not suitable for simultaneous polymer formation and biomolecule encapsulation.

The choice of the solvent in which the polymerization is carried out is also fundamental in terms of biological toxicity and biomolecule denaturation. For most hydrophilic monomers, including AAc and NIPAM, the polymerization can be carried out easily and with high yields in biocompatible solvents, such as H_2_O or DMSO. This implies that the formation of a hydrogel can be performed simultaneously with the encapsulation or tethering of a protein or a DNA strand. However, there is a wide range of monomers that are not soluble in water and, in order to be copolymerized in water-soluble backbones, other solvents must be used. Alcohols, such as EtOH or MeOH, stabilize the secondary structure of the protein (α-helix and β-hairpin), whilst leading to “molten globule” states as it denatures its tertiary structure. Since the biological or catalytic activity of a protein depends also on its integrity, the use of polar solvents during the synthesis of protein-polymer hybrid hydrogels is advised only if the tertiary structure is not relevant or when a thorough purification is carried out after the formation of hydrogels.

## 4. Introduction of Biomolecules within the Hydrogel Network

**Scheme 2:**
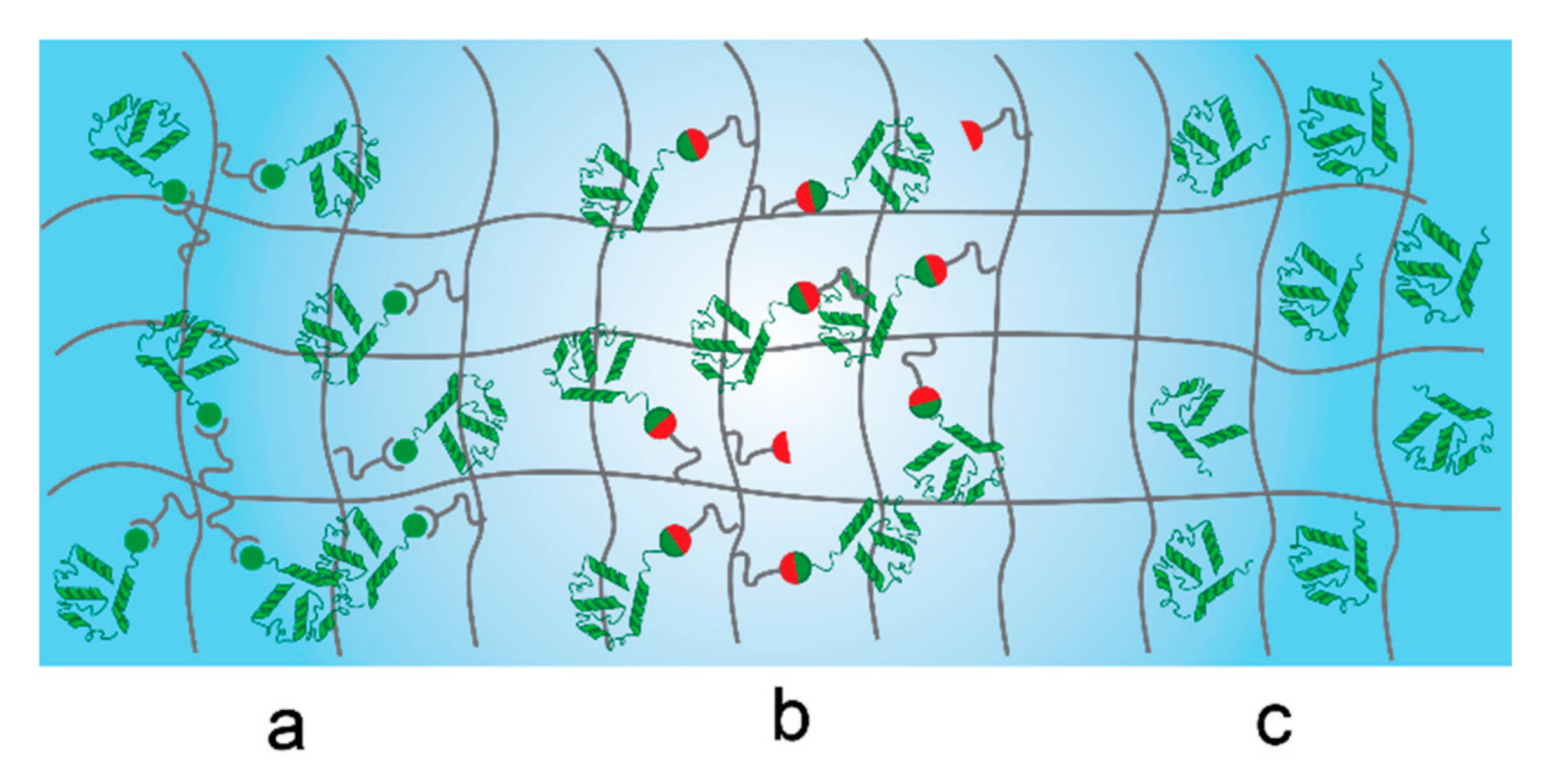
Schematic representation of the most common biomolecule immobilization approaches: (**a**) non-covalent interaction; (**b**) covalent attachment, and (**c**) physical entrapment.

The design of a bio-conjugated hydrogel scaffold depends on the specific application and also it has to be tailored to interact with the biomolecules incorporated within its structure. The entrapment and tethering of biomolecules for their subsequent release (or the release of their associated products) has brought a lot of interest in various biomedical research areas [166,167,168,169]. The incorporation of various biomolecules (Table 2) into the hydrogel matrix without affecting their conformation and activity is still challenging. Several strategies have been proposed for the encapsulation and attachment of heat-sensitive and fragile biomolecules [170], their selection depending on the nature of the biomolecule and the hydrogel structure, as presented below (Scheme 2).

Three general approaches have been used in the last years for biomolecule immobilization: covalent, non-covalent and physical entrapment. These approaches will be discussed below together with their role in the release of specific cargo molecules.

### 4.1. Covalent Tethering of Biomolecules

The immobilization of biomolecules by formation of covalent bonds on a network is one of the most widely used strategies because of the stable nature of the bonds formed between the hydrogel matrix and the biomolecules, serving to retain the biomolecules. A variety of covalent linkages have been explored for the attachment of biomolecules, including amide bonds formed through carbodiimide chemistry, thiol-ene bonds and other covalent bonds formed through metal-free click chemistry [185]. On the other hand, cleavable covalent linkages can be programmed to break over time or in response to a stimuli, ranging from small-molecule bonds, such as ester bonds and disulfide bonds, to macromolecular linkers, such as peptide and DNA sequences [186]. A special mention is the use of disulfide bonds because, even though a stable covalent bond is formed between matrix and biomolecules, this bond can be broken by the reaction with a suitable reducing agent, such as tris(2-carboxyethyl)phosphine, under mild conditions. In addition, since the reactivity of the thiol groups can be regulated by changing the pH, the yield of the methods involving disulfide bond formation is usually high [187,188] Moreover, immobilization of biomolecules can be also achieved in the pre-polymerization step: macromonomers can be composed of biomolecules. For example, four armed-PEG was linked with CMP to form hydrogels by triple helical interactions [189]. NHS-terminated PEG reacted with CMP either through single free amine at N-terminal or two free amines (one at the *N*-terminal and other at side of chain of lysine). Triple helixes by intramolecular interactions between CMPs at lower concentration and by intramolecular interactions between PEG-CMP conjugates at higher concentration could form. In addition to triple helixes, CMPs also worked as cross-linkers, leading to form chemically cross-linked network.

### 4.2. Supramolecular Tethering of Biomolecules

Several host-guest interactions make use of hydrophobic interactions for the attachment of biomolecules as a key approach in supramolecular hydrogels because of its dynamic nature [190]. The principle of affinity between complementary biomolecules has also been extensively applied to biomolecule bonding and release [191]. Macrocyclic oligosaccharides have an internal hydrophobic pocket with which hydrophobic drugs can associate. In those systems, the drug release is primarily controlled by the relative partitioning of solubilized drugs between the hydrogel and the release medium, and is independent of the mesh size of the hydrogel [192,193]. The high selectivity due to molecular recognition is a major advantage of host-guest interactions. Nevertheless, the use of host-guest chemistry often requires the covalent binding of a costly affinity ligand (e.g., antibody or aptamer) to the matrix [193]. Electrostatic interactions have been used to form strong non-covalent bonds between biomolecules and the polymer chains of the hydrogel [194]. The biomolecules are released when the hydrogel is degraded or when the electrostatic interaction is screened by the mobile ions from the environment. For example, a recombinant tissue inhibitor (rTIMP-3) was electrostatically entrapped within the matrix of a biodegradable scaffold, whose cross-links are degraded by metalloproteinase (MMP). When MMP attacks and digests the hydrogel cross-links, the consequent disruption of the network lead to the gradual rTIMP-3 release [195].

### 4.3. Uncontrolled Release of Biomolecules from Hydrogel Networks

Typical mesh sizes reported for hydrogels range from 5 to 100 nm [196]. Because of the network heterogeneity and polymer dispersity, hydrogels have a wide distribution of pore sizes within the same network, which allows the entrapment, transport and release of a cargo. The entrapment method is based on the retention of biomolecules within a hydrogel network that allows solvent molecules to pass through its porous structure but retains the biomolecules of interest. In general, the diffusion-dominated release of biomolecules cannot achieve over extended time points beyond a few hours to a day [197]. When the pore size approaches the biomolecule size, the effect of steric hindrance on biomolecules diffusion becomes dominant and the release rate slows down. A very interesting example is the release of GFs from hydrogels based on glycidyl methacrylated hyaluronic acid (HA-GM) and thiolated heparin (Hep-SH) that were 3D-printed in a core-shell bilayer architecture [198]. The large pore size of HA-GM-based hydrogels allowed free diffusion of the GF from the network. When heparin was co-incorporated into the matrix of hydrogels, the release rate was significantly decreased due to the electrostatic interactions between positively charged proteins (GFs) and the negatively charged heparin. By adding an external shell of HA-GM/Hep-SH hydrogel, the rate of GF release was controlled. If the pore size of the hydrogel is smaller than the biomolecule, this one remains entrapped inside of the network unless the network degrades, the attachment of the biomolecule breaks or the pores size is otherwise enlarged.

### 4.4. ‘On Demand’ Release of Biomolecules from Hydrogels Networks

The use of reversible methods to entrap biomolecules is highly attractive, mostly for economic reasons simply because when the biomolecule activity decays the matrix support can be regenerated and re-loaded with fresh biomolecules. For example, the reversible immobilization of enzymes is especially relevant for immobilizing labile enzymes and for applications in bioanalytical systems [199].

**Figure 4:**
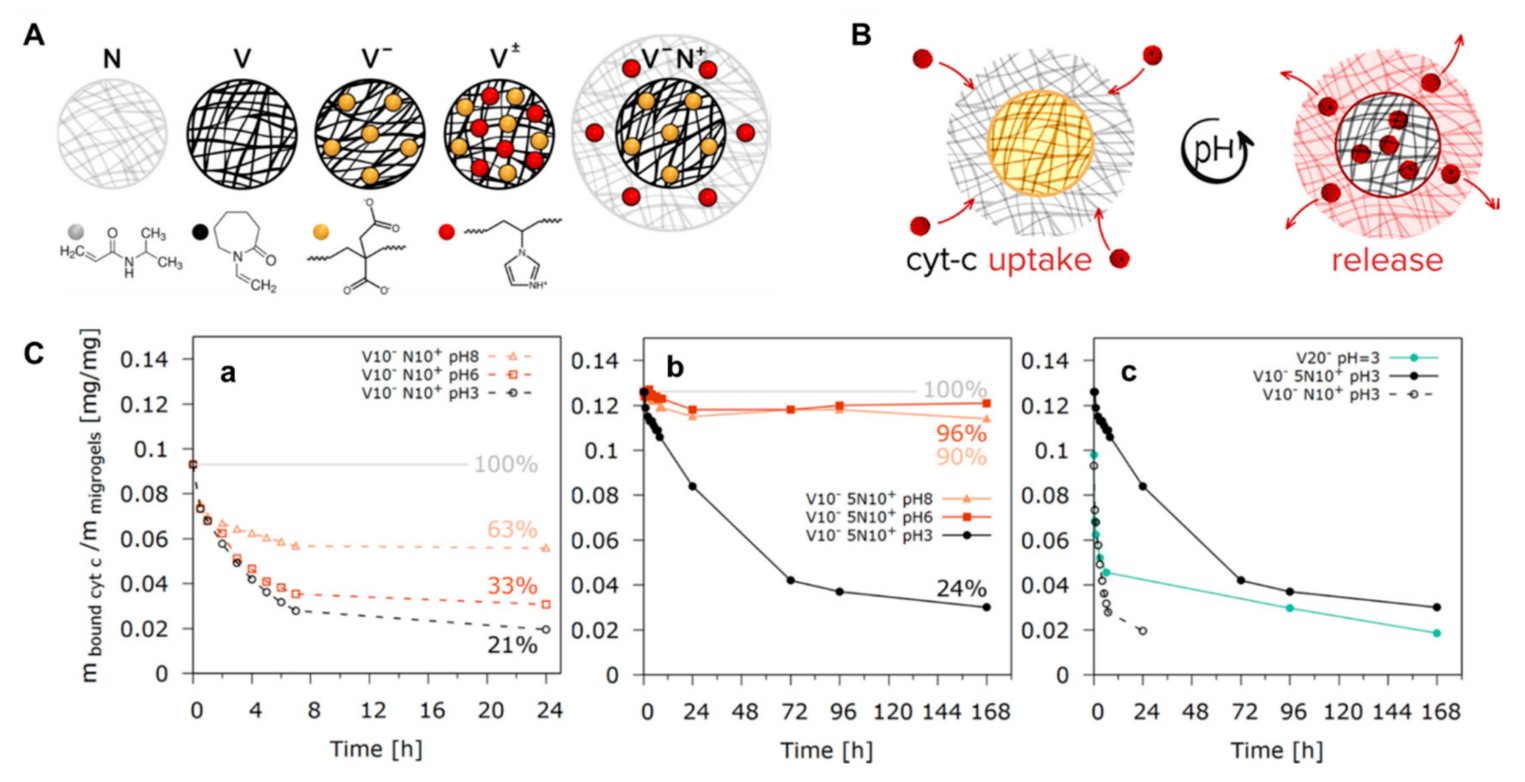
(**A**) Designing microgels for loading and release of the model protein, cytochrome c (cyt-c), by changing the distribution of ionizable groups into the microgel structure: *N*-pure PNIPAM microgel; V-pure PVCL microgel; V^−^-anionic PVCL microgel; V^±^-PVCL polyampholyte microgel with a random distribution of ionizable groups; V^−^N^+^-core−shell polyampholyte microgel consisting of an anionic PVCL core (black network) and a cationic PNIPAM shell (grey network); (**B**) Schematic representation of the protein loading and release into/from the polyampholyte microgels by reversibly changing the hydrogel charges from positive to negative using the pH switch from acidic to basic; (**C**) Effect of changing the pH value from 8 to 6, and respectively 3, on the amount of bound cyt-c for the polyampholyte microgels comprising of V10^−^N10^+^ (**a**) and V10^−^5N10^+^ (**b**); Variation of the amount of bound cyt-c vs. time at pH 3 for V10^−^N10^+^ and V10^−^5N10^+^ polyampholyte core-shell microgels in comparison to the V20^−^ polyelectrolyte microgels (**c**) [205].

Stimulus-responsive hydrogels have been especially useful, allowing for unprecedented levels of control over material properties in response to an external stimulus, thus improving treatment of many diseases and also improved approaches for tissue engineering and wound healing [200,201,202]. One strategy to control the release of biomolecules (or their products in the case of enzymes) initially entrapped in a hydrogel is to regulate network degradation. Degradation can occur either in the polymer backbone or at the cross-links. Some examples are the degradation mediated by the binding of biomolecule of interest. It has been reported an aptamer-cross-linked hydrogel trapped with glucoamylase [203]. Enzyme degradation is another important mechanism of action, multi-responsive P(MAAc-*co*-NVP) bio-conjugated hydrogels were synthesized with biodegradable, oligopeptide cross-links. This hydrogel was subject to enzyme-catalyzed degradation targeted by trypsin, for the purpose of drug delivery to the small intestine [204]. An additional method to release entrapped biomolecules consists in increasing the pore size by controlling the swelling of hydrogels. Another strategy to achieve control over the release from hydrogels is to use stimuli-responsive chemical bonds between the biomolecule and the polymer chains. This strategy is particularly relevant for small biomolecules that have to be released, because they would otherwise be released within a very short time period through spontaneous network diffusion (Figure 4) [205].

Thus, this approach was used to modulate the release of a model protein, cytochrome c (cyt-c), by controlling the content and distribution of acidic and basic moieties within the structure of polyampholyte microgels. Considering the ability of these microgels to reversibly switch their charges from positive to negative depending on the pH value, the microgel architecture was tuned to obtain a core-shell structure with an anionic itaconic acid-based PVCL core (black network) and a cationic PNIPAM shell (grey network) containing 1-vinylimidazole units. This shell acted as an electrostatic potential barrier controlling the release of the protein (Figure 4). Several chemical and physical interactions can be used alone or in combination, ranging from covalent conjugation to supramolecular interactions, such as electrostatic interactions and hydrophobic associations [26]. Finally, also physical methods such as ultrasound have been used in hydrogels for drug delivery and enhanced chemotherapy [206].

## 5. Biomolecules for Tissue Regeneration

Although easy-to-manipulate synthetic hydrogels have been prepared at a large scale for tissue engineering applications, they act only as passive scaffolds [207]. These hydrogels specifically lack from any specific bioactivities and do not support any cellular interactions [208,209]. Combination of synthetic polymers with biomolecules, such as nucleic acids (DNA and RNA), protein, carbohydrate and peptide, can be used to enhance the bioactivity of the resulted hydrogel matrix.

### 5.1. Nucleics Acids Conjugated to Synthetic Hydrogels

DNA and RNA are polynucleotides providing genetic information that can be delivered within the cell and stimulate differentiation and growth, but they can be also a biopolymer that can be used as a substitute building block for hydrogel-based assemblies [210,211] Compared to the natural and synthetic polymers, nucleic acids offer a high level of versatility and structural programmability for many biomedical applications. Therefore, there are significant efforts to enlarge the domain of nucleic acids-based hydrogels [31]. By attaching DNA to polymers as the side chain, DNAs function as non-covalent cross-linkers in the hydrogel network and support the self-assembling of the synthetic networks into second-order or higher-ordered structures in suitable environments, leading to form multifunctional materials based on DNAs and non-natural polymers [171,212,213]. DNA-hydrogel assemblies can be formed with high mechanical strength, biocompatibility and controllable morphologies while their functions, such as sequence designability, recognition ability, responsiveness, and programmability can be easily adjusted by the choice of DNA and hydrogel [214]. DNA-linked hydrogels are a great alternative, to classically cross-linked gels for cell scaffolding [31,215]. Besides the removal of the generally toxic cross-linker, DNA gels resemble perfectly the ECM, and make progressively space for cells to growth. Based on their composition, DNA-hydrogels are divided into two groups; (i) pure DNA supramolecular hydrogels which are formed by branched and linear DNA assembled matrixes depending on the design criteria and (ii) hybrid DNA supramolecular hydrogels which employ synthetic polymers as backbone and DNAs as switchable cross-linking points [173].

**Scheme 3:**
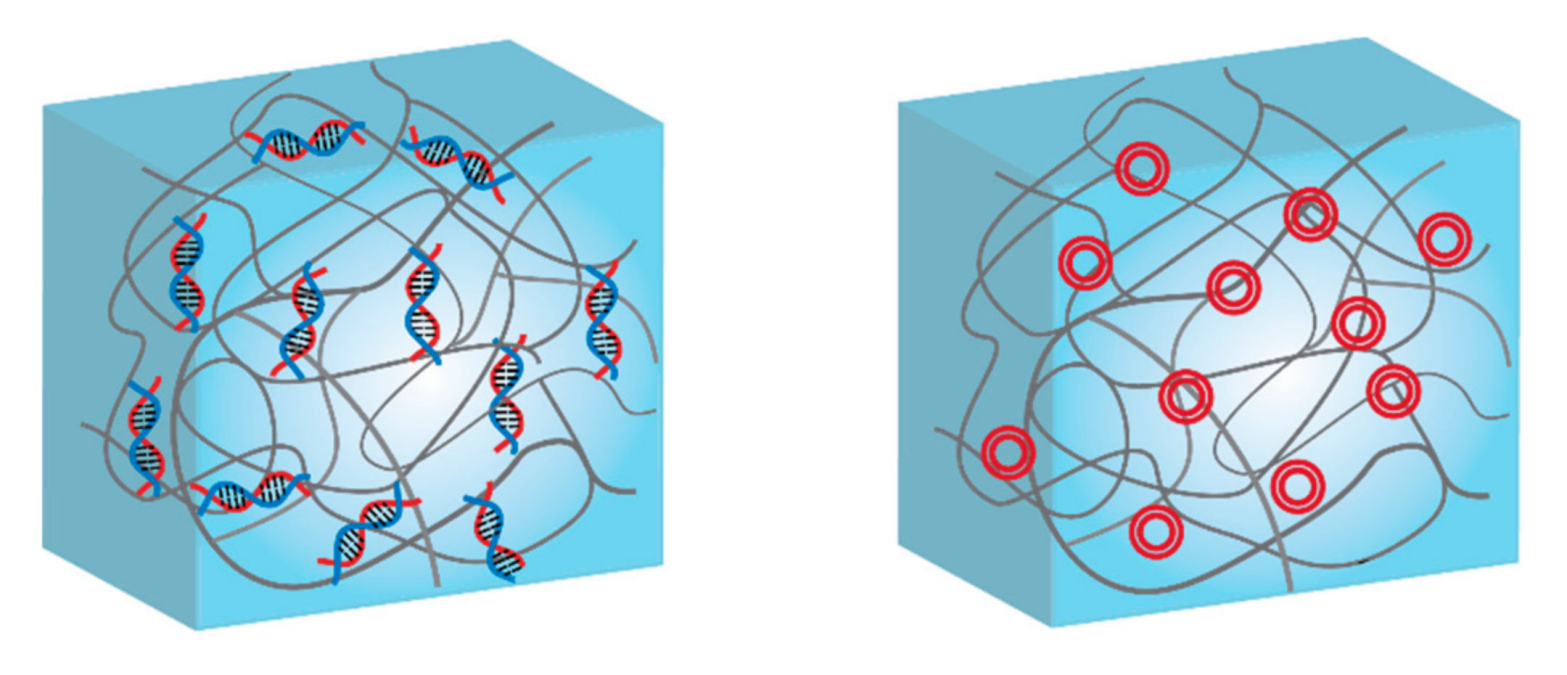
Schematics of DNA-functionalized hybrid hydrogels: (left) single-stranded DNA covalently attached to the polymer scaffold, and (right) plasmids or long DNA sequences conjugated by electrostatic interactions.

Generally, DNA is attached covalently on the polymeric chain in order to function as a stable cross-linking agent [126]. The covalent bond is generated by free-radical polymerization [216,217], click chemistry [218], and thiol-ene chemistry [219]. Over all these reactions, click chemistry has been mainly used since it is not only fast, specific and efficient [220], but also permits selective modification and attachment of biologically active entities (e.g., adhesion or enzymatically degradable peptides) [221,222]. Besides chemical conjugation, noncovalent interactions (e.g., electrostatic interaction [223], hydrophobic interactions [224], hydrogen bonds [225] and π−π interaction [226] between DNA and polymer) have been employed (Scheme 3). The main advantages of hydrogels prepared by noncovalent interactions are that they can form interconnected networks without need of toxic chemicals, bioactive molecules can be easily entrapped, and they are biocompatible as medical biomaterials [174]. For example, double-stranded DNAs were mixed with positively charged, water-soluble poly(phenylenevinylene) to form a physical hydrogel [227]. Such system was proposed to track the drug release since the polymer used made the hydrogel fluorescent.

As the first example of hybrid DNA-hydrogels, the 5′-terminal-amino-modified single stranded DNA (ssDNA) chains were conjugated with a P(DMAM-*co*-NAS) poly (*N*,*N*-dimethylacrylamide-*co*-*N*-acryloyloxysuccinimide) through an active ester-amine click reaction. Then ssDNAs were cross-linked with complementary DNA strands to form three-dimensional (3D) polymer network [228]. Simply, individual ssDNAs were incorporated into polyacrylamide to obtain either oligoT- or oligoA-polyacrylamide and then they cross-linked with each other to form hybrid DNA-polyacrylamide hydrogels. In this hydrogel, DNA cross-linking was reversible depending on the change of temperature, leading to use such system for bio sensing applications. Although DNA cross-linking has been mainly used for many of DNA-hydrogel systems, resulting assemblies suffer from poor permeability of nutrients (e.g., larger proteins in size) due to the presence of small mesh in hydrogel network [229]. This limits to use DNA-hydrogels in 3D cell culture and tissue engineering applications. Besides being a functional cross-linker, DNA is also employed as a molecular information carrier that sends growth impulses to the tissue. In order to deliver the information to the cells in the damaged tissue and promote the healing of it, this has to enter the cell nucleus, and due to its size and change the delivery is highly inefficient. There are few examples of efficient delivery, where DNA is delivered under the form of a plasmid with and encoded GF [230].

Furthermore, RNAs in the form of short-interfering RNAs and microRNAs associated with hydrogels have been successfully applied in the treatment of various diseases, in particular cancer and infections [231]. Besides, the use of non-coding RNAs in molecular recognition and tissue engineering has been also extended towards a new target, mainly the in vitro and in vivo tissue growth [232]. More RNA-hydrogel based systems have been reviewed in more detail elsewhere [45,233,234].

### 5.2. Proteins Conjugated to Synthetic Networks

Recently, biocompatible and biodegradable protein hydrogels have been developed to be used as functional biomaterials in the field of therapeutic delivery and tissue engineering. For example, there are synthetic protein based hydrogels which are derived from single protein component such as elastin [175], collagen [176], and globular proteins (e.g., bovine serum albumin, ovalbumin and YajC-CT) [177,235]. The main advantages of pure protein hydrogels are relatively homogenous network structure and simple procedure. However, often recombinant proteins are not only expensive and difficult to produce at high amounts but also are not soluble in water and denatured during the formation of protein hydrogels.

**Figure 5:**
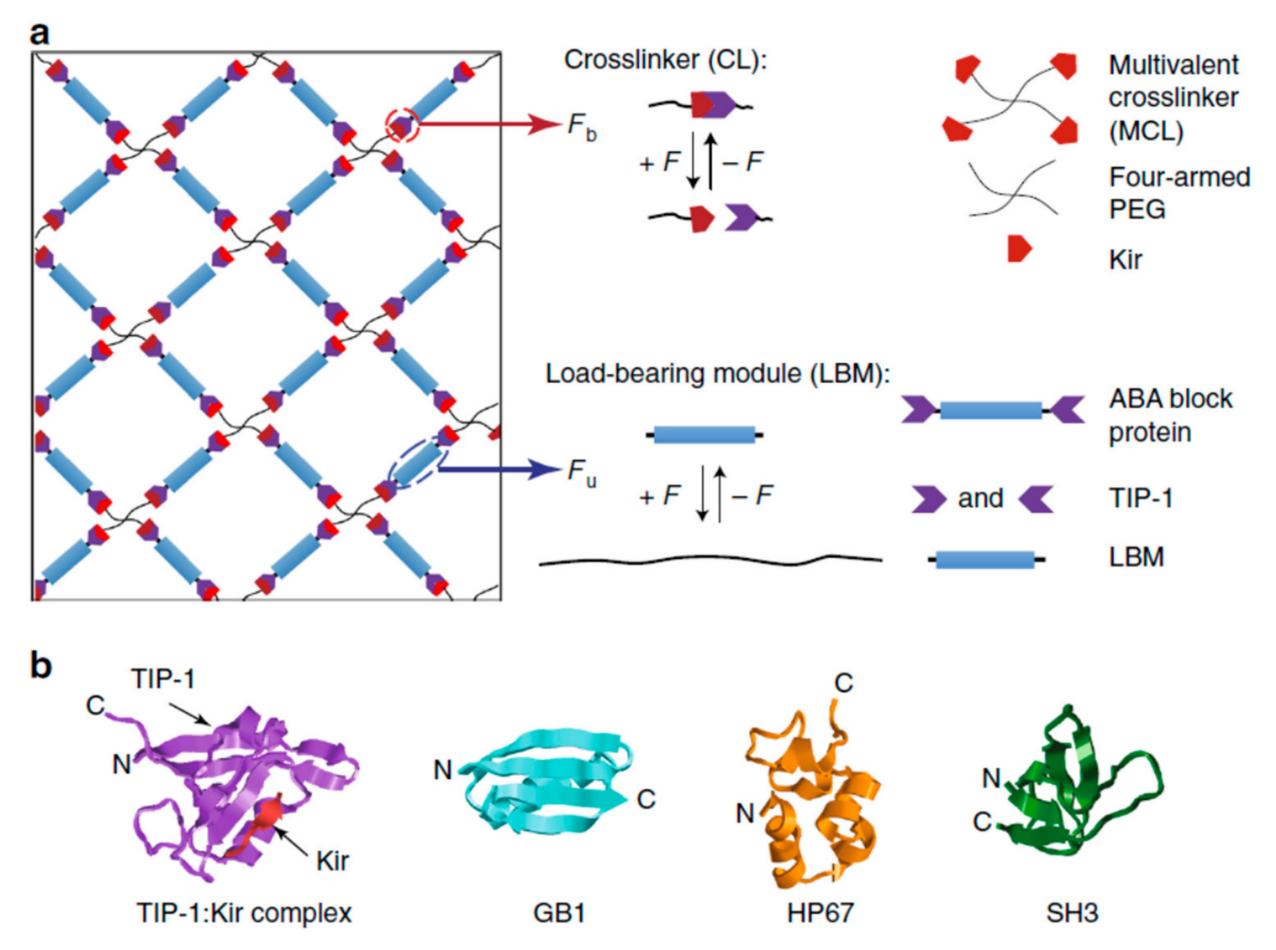
Design of hydrogel networks with mechanical properties similar to naturally occurring tissues: (**a**) The hydrogel network is formed from two mechanical responsive elements: cross-linkers (CLs) and load-bearing modules (LBMs). The binding/unbinding kinetics of the CL and the folding/unfolding of the LBM are regulated by the applied mechanical force; (**b**) Structure of the protein domains (LBMs) used in the hydrogel design to form the TIP-1:Kir complex: GB1, HP67, and SH3 [178]. The figure is reproduced from an open access article published by Nature under a Creative Commons Attribution 4.0 International License (https://www.nature.com/articles/s41467-018-02917-6#rightslink).

**Figure 6:**
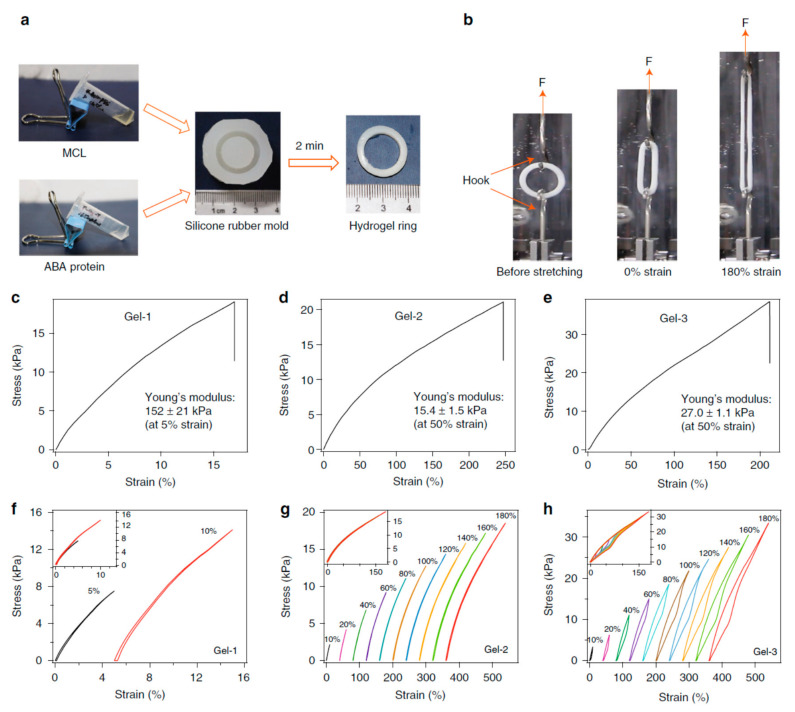
The bulk mechanical properties of the designed hydrogels: (**a**) General procedure for preparation of protein hydrogel rings for mechanical testing. All protein gels were cross-linked using TIP-1:Kir complexes. (GB1)8, (GB1-HP67)4 and SH3 were used as the LBMs and the corresponding hydrogels were denoted as Gel-1, Gel-2 and Gel-3, respectively; (**b**) Setup for the mechanical tests of ring-shaped hydrogel. Stress-strain curves for the Gel-1, Gel-2 and Gel-3 until their breakage (**c**–**e**). Representative stretching-relaxation curves for the Gel-1, Gel-2 and Gel-3 (**f**–**h**). The curves are horizontally offset for clarity. The final strains are shown on the curves. Insets show the superposition of the stretching-relaxation curves at different strains [178]. The figure is reproduced from an open access article published by Nature under a Creative Commons Attribution 4.0 International License (https://www.nature.com/articles/s41467-018-02917-6#rightslink).

In protein-conjugated hydrogel networks, the proteins can be either cross-linkers, load-bearing entities or molecular cues [181,236]. Cross-linker serve as a force transductor between different protein chains whereas load-bearing entities regulate mechanical response of individual protein chains. Depending on the cross-linking mechanism, the resulting hydrogels named as dynamic physical gels and stable chemical (or covalent) gels [178,237,238,239]. The structure and properties of the primary chains along with cross-linking density contribute to the chemical gels whereas molecular entanglement and secondary molecular interactions play role in the formation of the physical gels. Physical gels are often reversible and the interactions that hold physical gels can be disrupted by changes in environment such as temperature, pH, ionic strength and so on. Besides, for hybrid protein-polymer hydrogels, load bearing molecules are responsible for the deformation of the network and the hydrogel fracture occurs due to breaking of cross-linkers [233]. When the hydrogel is cross-linked by specific ligand-receptor complexation and global proteins serve as load-bearing molecules, the complex mechanical response of hydrogel network is driven by both unfolding/refolding of global proteins and reversible bond rupture/reformation of ligand-receptor complexation [181]. Thus, depending on the mechanical properties of cross-linkers and load bearing molecules, the mechanical properties of hydrogel network can be predicted. Towards this goal, three different hydrogels with predicted mechanical response have been designed [178]. The hydrogels were composed of two components: (i) a multivalent cross-linker (MCL), which consists of a four-armed PEG macromolecule fused with four Kir2.3 C-terminal tail peptides (Kir), and (ii) ABA-type block proteins, which are composed of two Tax-interacting protein-1 (TIP-1) domains at the both ends of various proteins with distinct mechanical properties, namely GB1, HP67, and SH3 respectively (Figure 5). The TIP-1 interacts with the Kir peptide forming a complex with a high binding affinity, and this complexation acts as cross-linker (CL), whereas the center of the ABA block protein is the load-bearing molecule (LBM). The mechanical properties of individual cross-linker and load-bearing molecule were characterized by single-molecule force spectroscopy and it was found out that the designed hydrogels exhibited the expected mechanical properties on a molecular level. Then, synthetic protein hydrogels were prepared for mechanical testing in PBS at RT (Figure 6). According to the stress-strain curves, Gel-1 was only extended by approximately 16% with Young’s modulus of about 150 kPa at a strain of 5% whereas Gel-2 was extended by 250% with Young’s modulus of about 15 kPa at a strain of 50%, and Gel-3 was highly extensible with a failure strain of ~215% and a Young’s modulus of ~27 kPa at a strain of 50%. These findings offer a framework for construction of protein hydrogels with defined mechanical properties (e.g., elasticity, extensibility, toughness and self-healing) for biomedical applications.

**Figure 7:**
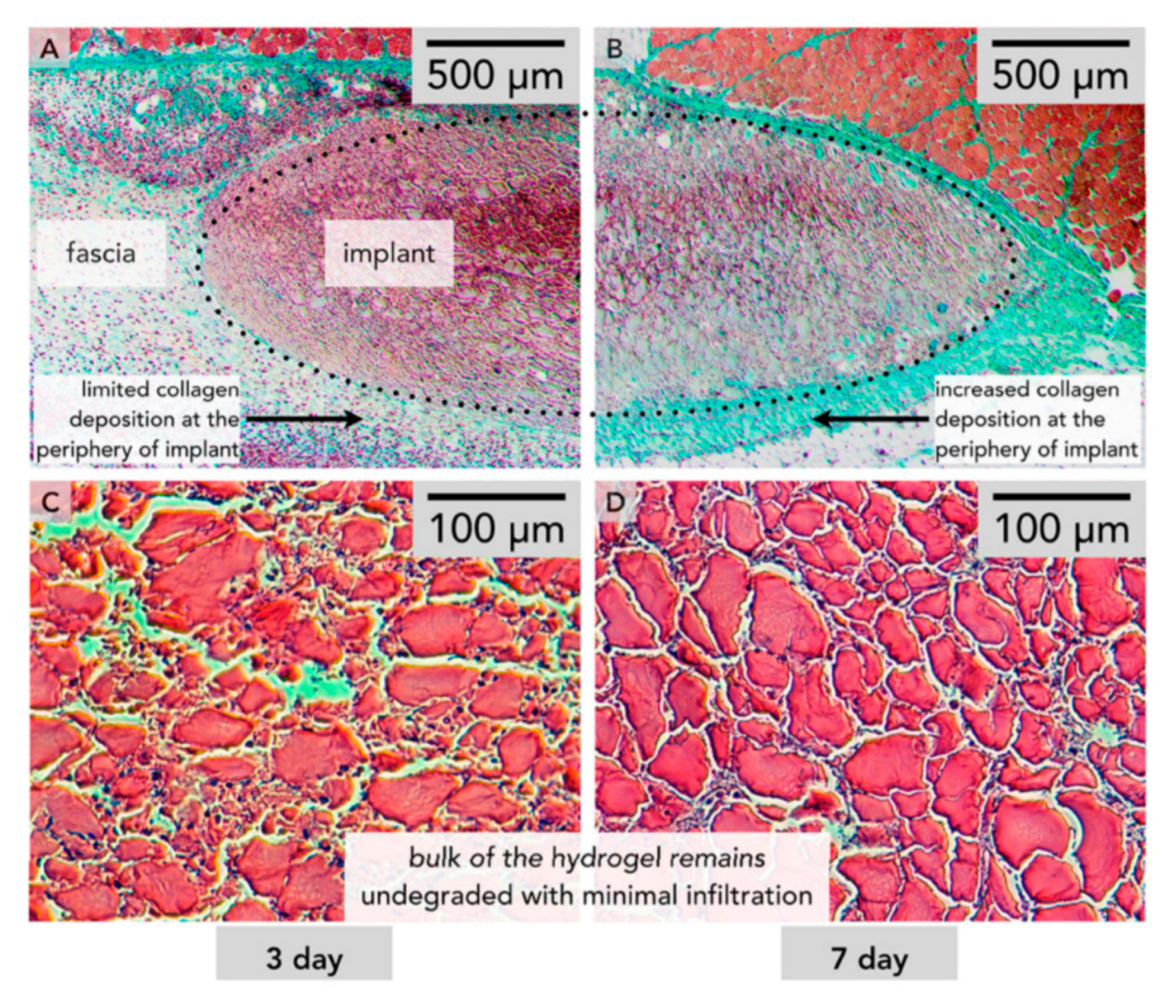
Histology of a self-assembled peptide hydrogel with mMA encoded within after 3 days (**A**,**C**) and after 7 days (**B**,**D**). (**C**) and (**D**) show close to no cellular growth within the gel matrix [257].

Furthermore, to engineer protein-polymer hybrid hydrogels, cysteine residues have been used to be cross-linked according to the thiol-Michael addition reactions (e.g., with electron-deficient carbon-carbon double bonds or the free radical thiol-ene reaction via electron-rich/poor carbon-carbon double bonds) [240,241]. Electron-deficient olefins for thiol-Michael addition reactions can be applied for methacrylates, acrylates/acrylamides, vinyl sulfones, and maleimides [242]. However, these reactions suffer from stability of cysteine residues without oxidation and slow reaction rate to create homogenous hydrogels [243]. Besides, thiol-ene reactions have been combined with photo-initiated free radical production, leading to spatiotemporally controllable hydrogel production [241,244]. Nevertheless, this type of reaction competes with homo-polymerization of enes and therefore hydrogel production must be done in caution [245]. Additionally, genetically encodable click chemistries (e.g., SpyCatcher/tag, SNAP-tag, Snoop-tag, Halo-tag and CLIP-tag) have been applied to engineer protein hydrogels [161,246,247,248,249,250]. For example, two peptide fragments derived from the CnaB2 domain of Streptococcus pyogenes were covalently bound to each other via SpyCatcher/tag chemistry, resulting to pure protein hydrogels [251]. In parallel with the development of multifunctional hydrogels from polymer and recombinant proteins, functional proteins such as enzymes and antibodies have been also incorporated into the polymer network. For example, calmodulin was incorporated either into cross-linked polyacrylamide (PA) or PEG polymer networks, leading to calcium responsive hydrogel [252,253]. Both hydrogel network underwent construction in the presence of calcium ions and swell upon its chelation. There are also similar hydrogels that swell and contract due to antigen-antibody binding and catalytic activity of grafted enzymes [254,255]. When the protein becomes the bioactive element in the gel, then it transports an information towards the cell. Proteins that carry information are generally zinc finger proteins (ZFPs), TALENS and monoclonal antibodies. ZFPs and TALENS can recognize and bind DNA pairs, and block the expression of a gene. They are particularly useful to target cancer cells and silence them [256]. More used are mAB which bind to an antigen to inactivate it or stimulate and immune response towards it [256]. Thus, instead of promoting cell growth and regeneration, mAB are used to actively deplete the growth of a tissue, for instance to fight the formation of excessive blood vessels in the retina, which leads to eyesight loss. These mAB were encoded within in a synthetic self-assembled peptide gel, and depending on the amount of gel present, the degree of vascularization could be inhibited accordingly (Figure 7) [257].

Another way to deliver mAB is to tether them non-covalently on a hydrogel network, and tune their release by introducing a competition molecule. This is the case for a poly(carboxybetaine) hydrogel decorated with streptavidin molecules. These can bind the mAB, with however a lower affinity strength as towards biotin. Once biotin was introduced, the mAB could be released, depending on the biotin concentration, leading to a sustained release material [258].

Moreover, hydrogels can be also loaded with different GFs for promoting rapid skin tissue regeneration and improve the angiogenesis, reepithelization, and neovascularization within the damaged tissues [259]. For example, hydrogels have been loaded with different GFs, including granule-lyophilised platelet rich fibrin [260], vascular endothelial GFs [260], collagen I [261], bone morphogenic proteins [262], basic fibroblast GF [263]. More specifically, a PVA-based hydrogel was loaded with granule-lypohilised platelet rich fibrin for treatment of skin wounds. PVA improved the hydrogel biodegradability and mechanical properties whereas the GFs promoted faster rate of wound healing with enhanced neovascularization and reepithelization. In addition, collagen I, known to play a key role in smooth muscle formation and reepithelization of damaged skin, was conjugated to various hydrogels with for soft tissue engineering applications [261].

### 5.3. Carbohydrates Conjugated to Synthetic Hydrogels

**Figure 8:**
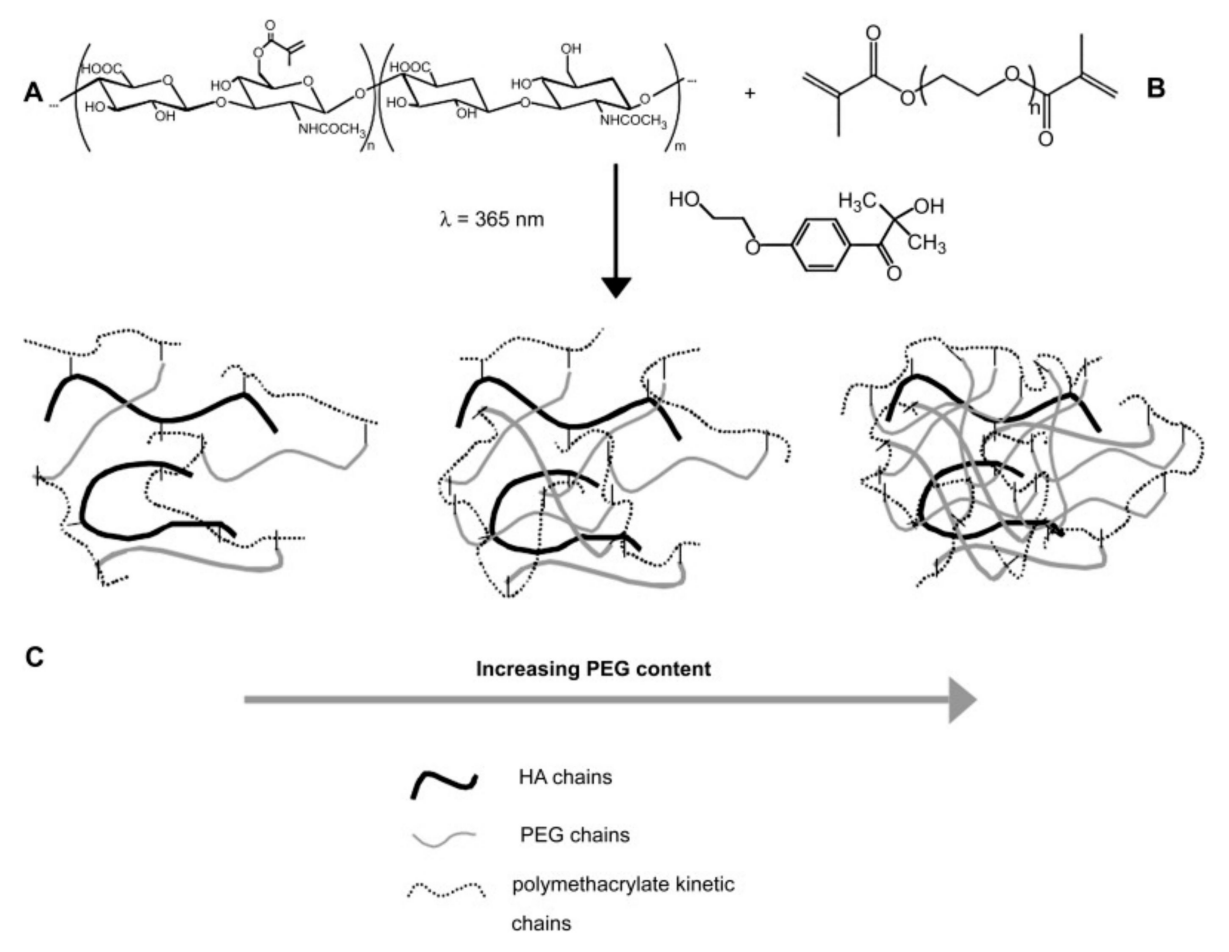
(**A**) Multimethacrylated HA with m + n (m + n ≈ 10,000) repeat units (Mr ≈ 400 Da). In this study, ~1 in 5 disaccharide units was functionalized with a methacrylate group. (**B**) Dimethacrylated PEG chains with n repeat units (Mr ≈ 44, n ≈ 105) were end-capped with methacrylate groups. (**C**) Hydrogel networks formed by radical initiated chain copolymerization of HA with varying amounts of PEG. Primary radicals were produced by the dissociation of the photoinitiator, 1-[4-(2-hydroxyethoxy)-phenyl]-2-hydroxy-2-methyl-1-propanone, in the presence of UV light (l = 365 nm) [180].

Carbohydrates, such as glycosaminoglycans (GAGs) and hyaluronic acid (HA), as components of the ECM, are known for a wide range of biological functions. However, GAGs show chemical and structural heterogeneity and are difficult to produce, which limits their use in hydrogel-based systems. On the other hand, HA, with a simple and homogenous molecular structure composed of β-1,4-linked d-glucuronic acid and β-1,3-*N*-acetyl-d-glucosamine disaccharide units, it is easy to produce [264]. Additionally, HA plays an important role in cellular adhesion, signaling differentiation, wound healing and morphogenesis [179]. For example, ECMs based on hyaluronan derivatives and PEGDA were used to form covalently cross-linked and biodegradable 3D hydrogels for culture of primary and stem cells in vitro and tissue formation in vivo [265]. In another example, valvular interstitial cells (VICs) were encapsulated in enzymatically degradable and cross-linked 3D hydrogel cell culture platforms, which were made by photo-initiated chain copolymerization of a methacrylate-containing HA with a PEG cross-linker (Figure 8) [180]. Particularly, the increase of PEG content in hydrogels caused a delay or prevented reverse gelation, while increasing the HA content led to a much faster degradation. Moreover, the HA fragments released into the cell culture medium contributed to the manipulation of the secretory properties of encapsulated VICs. The dual functionality shown by this bio-conjugated hydrogel recommends it as a very promising material for heart valve tissue engineering.

### 5.4. Peptides Conjugated to Synthetic Hydrogels

**Scheme 4:**
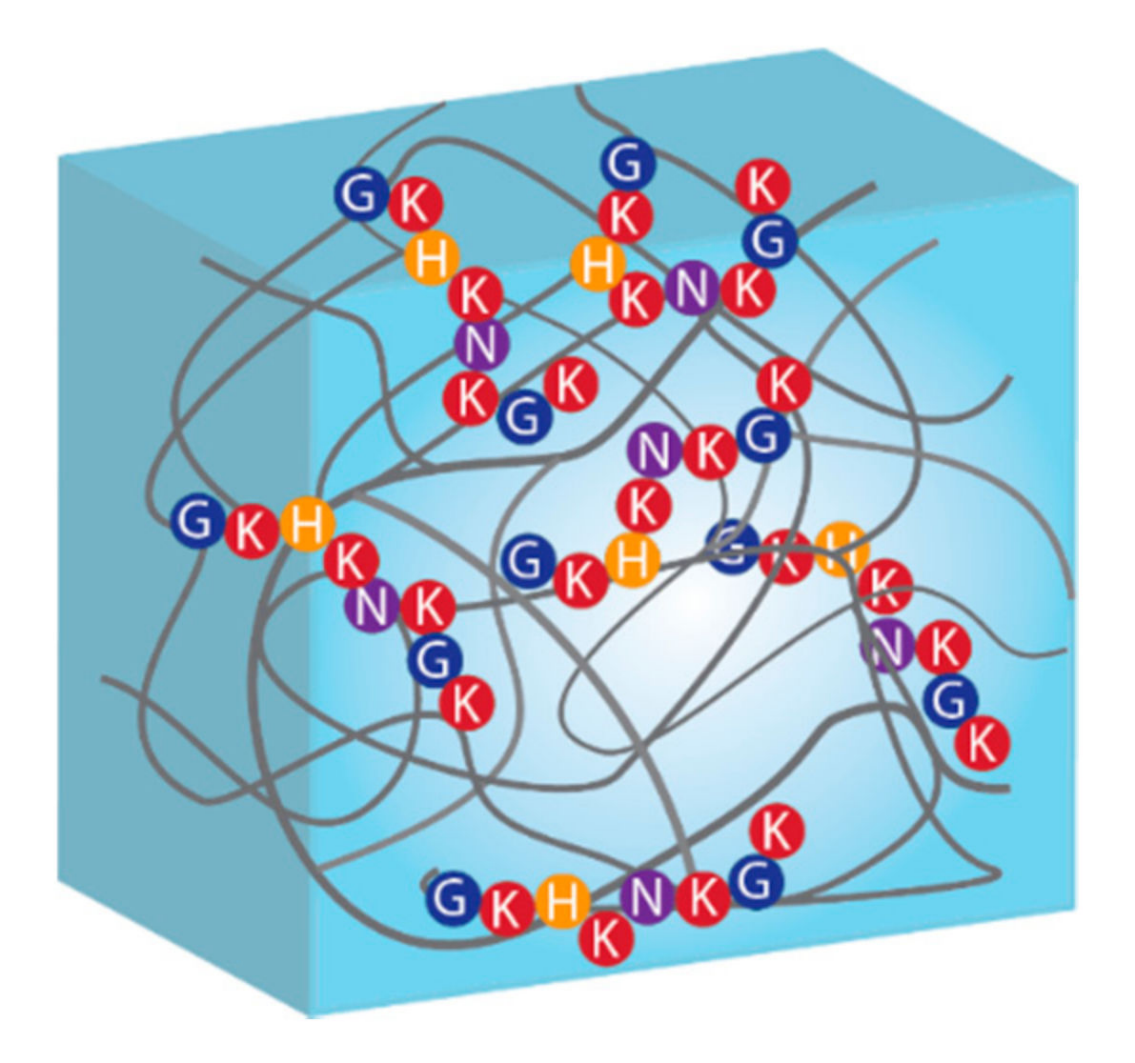
Schematic representation of peptide-functionalized hybrid hydrogels.

Peptide-hydrogels are prepared by the self-assembly of oligopeptides into fibrous nanostructures that generate gels due to cross-linking based on non-covalent interactions and entanglements (Scheme 4).

**Figure 9:**
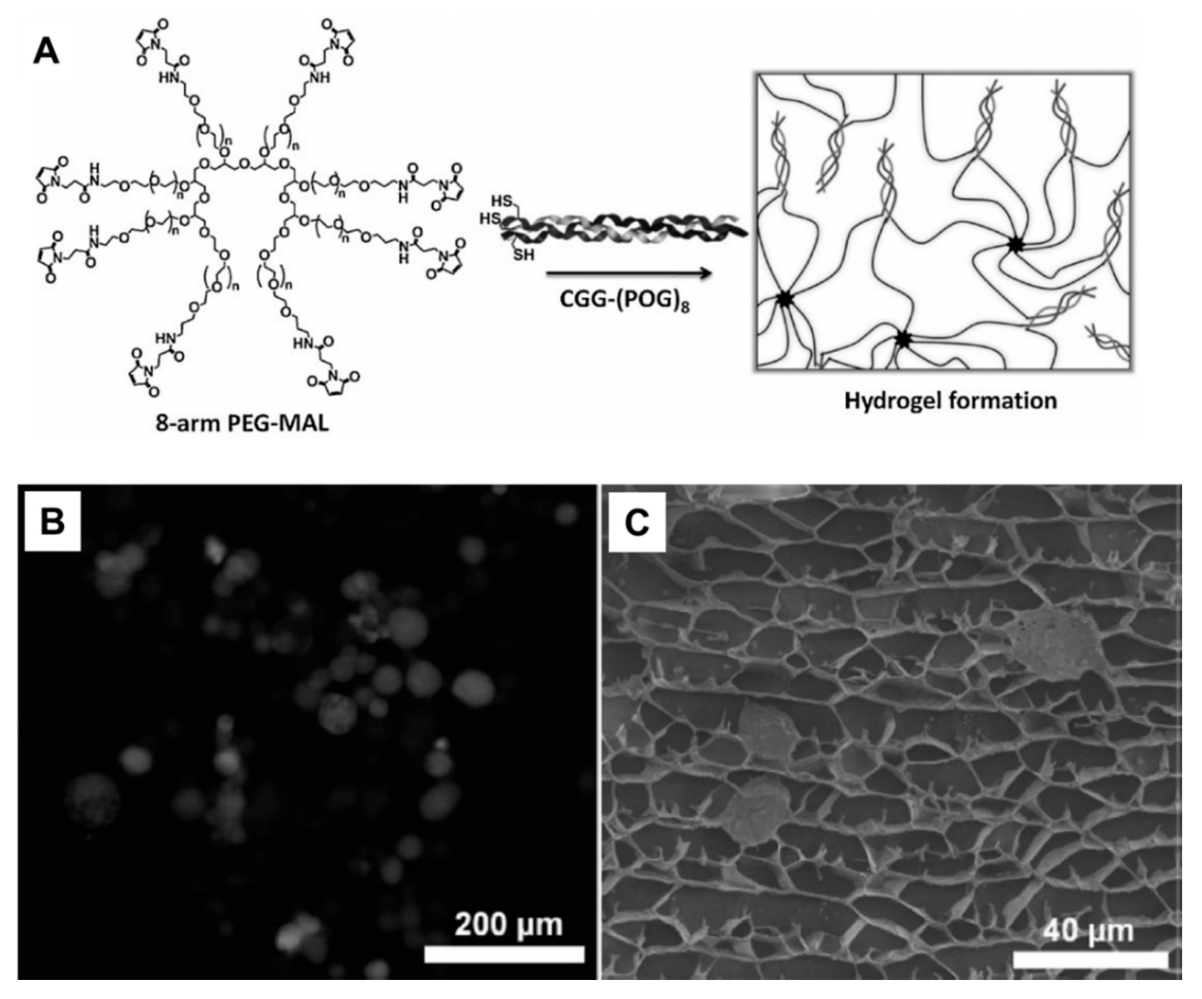
(**A**) Fluorescence image of hMSC cells stained with Calcein, entrapped within the non-stained gel matrix. (**B**) Cryo-SEM image of hMSC entrapped within the gel pores. (**C**) Scheme of hydrogel formation via the reaction between an 8-arm maleimide functional PEG and a collagen triple helical peptide [182].

Typical pure peptide hydrogels are formed by supramolecular fibers of the β-sheet motifs and gelation of α-helical coiled coils and collagen mimetic polyproline helices which have been reviewed elsewhere [28,266,267,268]. Hybrid peptide-polymer hydrogels are formed by either direct self-assembly of synthetic polymer chains conjugated with peptides or direct co-assembly with polymers to produce supramolecular hydrogels [269]. PEG is very suitable as basis in the design of hybrid peptide-polymer hydrogels because it enhances the circulation time of peptides when they are conjugated to PEG [183,184]. One way to attach PEG to peptides is to utilize a multi-arm PEG to lead to a network. Attachment of enzymatically degradable peptide brings biodegradability whereas heparin-binding peptides provide biocompability [181,270]. Stiffness of resulting hydrogels were in the range of physically cross-linked systems and thus PEG-CMP hydrogels can be used as soft substrates for cell-culture applications (Figure 9). In other examples, cysteine modified CMPs were conjugated to multi-armed PEG through maleimide functional group [182]. Resulting hydrogels showed thermo-responsive behavior and changeable mechanical properties depending on CMP length. Besides, these hydrogels also exhibited honeycomb-like structure with pore size of 5–25 µm and used as 3D environments for culture of human mesenchymal stem cells (hMSCs). The PEG was also used to produce elastin-based hydrogels [183]. A peptide sequence (X(AKAAAKA)2X) was attached to the azide functionalized polymer using copper(I) catalyzed azide-alkyne cycloaddition (aka click reaction) and then hexamethylene diisocyanate was used to induce cross-linking and further gelation. Several other polymers including PPO [183], PEODA [271], and HPMA [272], PNIPAM [273] have been also applied as hydrogel scaffolds. Instead of only PEG, Pluronics (PEG PPO-PEG block copolymer) were functionalized with peptide (AKAAAKA)_2_ and self-assembled into micelles and formed stable viscoelastic hydrogels [183]. Besides, collagen mimetic peptide ((Pro-Hyp-Gly)_7_-Tyr) was coupled to backbone of PEODA and resulting hydrogels were used as tissue engineering scaffolds [271]. In another example, a linear copolymer was synthesized by radical copolymerization of HPMA and the metal-chelating monomer DAMA [272]. Two histidine tagged peptides (Coiled Coil (CC) 1 peptide, the amino acid region Val336-Ser590 from the Drosophila kinesin stalk protein and CC2 peptide, the de novo designed coiled-coil sequence EK42) were separately mixed with the polymer in the presence of nickel ions. Histidine-tagged CC peptides were formed complexes with iminodiacetate groups in the presence of nickel ions, leading to form temperature responsive hybrid peptide-polymer hydrogels. In another example of thermo-responsive hybrid hydrogels, PNIPAM has been conjugated with a short ionic complementary peptide (FEFEFKFK) [273], the LLEELLEELEELLEEA (LE) peptide which forms α-helix structure in the presence of calcium ions [273].

Co-assembled peptide-polymer hydrogels show high stability, tuneable mechanical properties, better biofunctionality and develop new architectural versatility compared to the hybrid hydrogels based on the self-assembly of peptide-conjugated polymers [274]. For example, low molecular weight hydrogelator Na-fluorenylmethyloxycarbonyl diphenylalanine (Fmoc-FF) and hexapeptide based soft hydrogelator PEG_8_-(FY)3 with three repetitions of the Phe-Tyr units and a *N*-terminus PEG moiety was co-assembled to form biocompatible hydrogels [275]. Rheology analysis confirmed the improved mechanical properties of co-assembled hybrid gels. Besides, the presence of PEG slowed down the gel kinetic formation from 42 to 18 min and reduced the gel rigidity (reported as storage modulus, *G’*) from 9 to 6 kPa, leading to be exogenous scaffold materials for tissue engineering applications. In another example, peptide based co-assembled hydrogels consisted of two building blocks (Fmoc-FF and Fmoc-arginine (Fmoc-R)), followed by incorporation of hydroxyapatite into the hydrogel matrix, resulting into 3D hydrogel scaffolds for bone tissue regeneration [276].

## 6. Conclusions and Future Perspectives

The design of synthetic materials for regenerative medicine poses a series of challenges in terms of biocompatibility, mechanical resistance and adaptability. The coupling of bio-inert polymer networks with cell-active biomolecules requires the precise selection of hydrogel synthetic path and biomolecule conjugation conditions to preserve their integrity and bioactivity. Hence, the interplay between functionality and bioactivity relies on the careful choice of polymer structure, synthetic, production path and specific biomolecule. Here, we addressed recent advances in conjugation of the most common biomolecules (nucleic acids, proteins, short peptide sequences and carbohydrates) to functional hydrogel soft-materials for regenerative medicine purposes. In addition, several applications of bio-functionalized hydrogels were discussed, including the development of in vitro scaffolds, injectable degradable scaffolds, wound healing, cartilage implants and gene delivery applications.

While numerous developments have been made so far in the area of bio-conjugated hydrogels for biomedical applications, important challenges are still to be overcome. For example, further improvements in the synthesis and structure of hydrogel matrices should be done to reduce the immune response, and thus to minimize the inflammatory processes. Besides this, the exploitation of multistimuli-responsive components would increase the functionality and versatility of bio-conjugated hydrogel, widening their application range. The stimuli-responsive control over the stiffness of hydrogels could be used to synthesize bio-material interfaces for the guided growth of cells and, specifically, the differentiation of stem cells. Even though there is a plethora of examples of hydrogels conjugated to single biomolecules, there are only few examples of hydrogels that contain multiple types of biomolecules working together to serve the same purpose. In fact, integration of several biomolecules into one hydrogel system could lead to the development of new materials in which cascade reactions can take place by integration of enzyme cofactors. As a consequence, these bio-materials are expected to continuously draw a growing scientific and technological attention towards translational applications.

## Figures and Tables

**Table 1:** The most relevant polymers involved in preparation of hydrogels for biomedical applications.

Polymer	Abbreviation	Structure	Properties	Applications	Refs.
Poly(2-hydroxyethyl methacrylate)	PHEMA	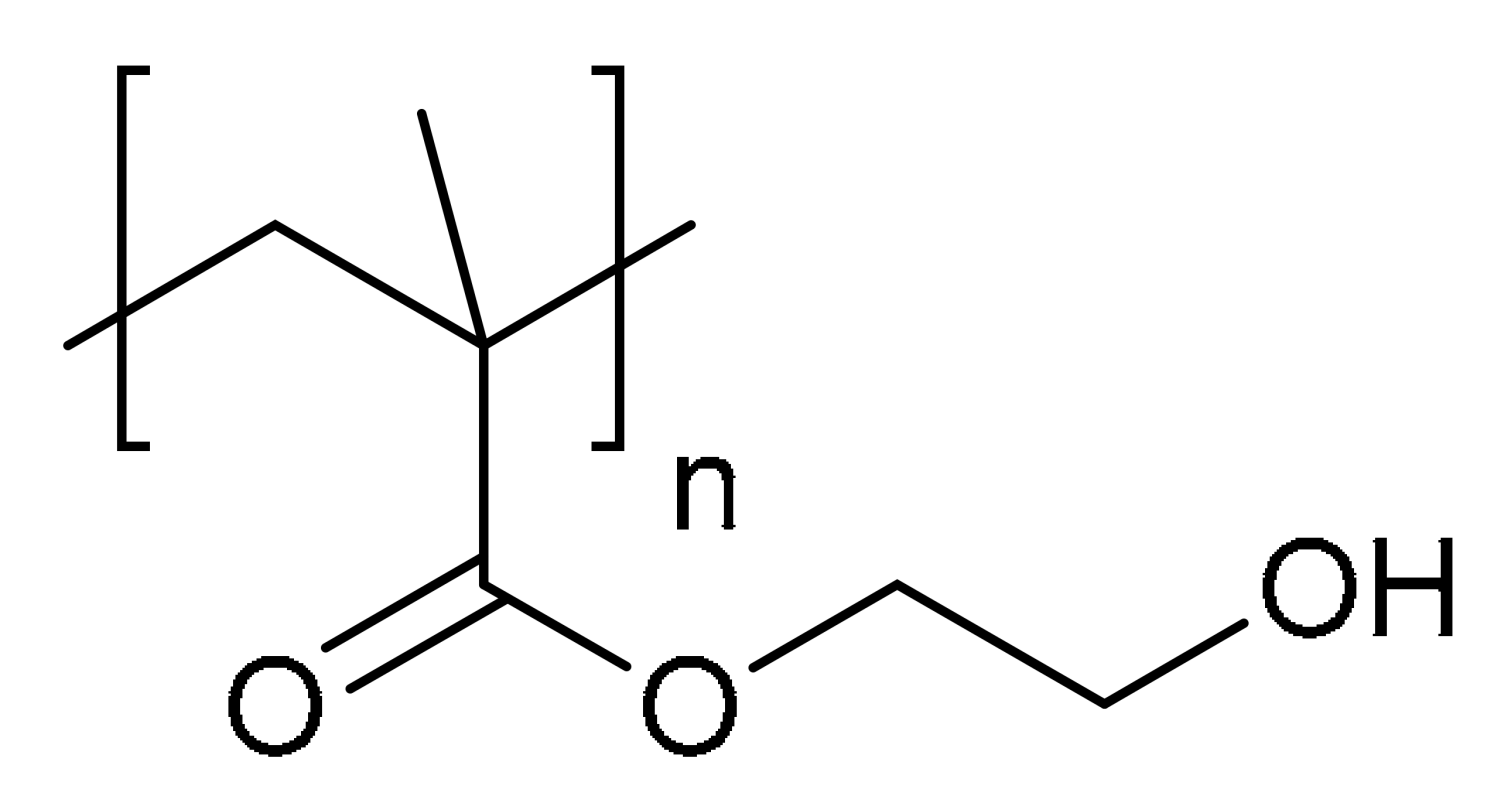	Hydrophilic, biocompatible	cell growth; tissue engineering; regenerative medicine	[134,135,136]
Poly(2-hydroxypropyl methacrylate)	PHPMA	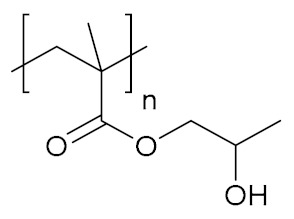	Hydrophilic, biocompatible	cell growth; tissue regeneration	[125]
Poly(ethylene glycol)/poly(ethylene oxide)	PEG/PEO	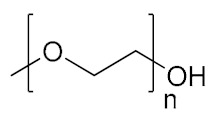	Hydrophilic, biocompatible	cell growth; tissue engineering; regenerative medicine	[137,138]
Poly(2-methyl-2-oxazoline)	PMOXA	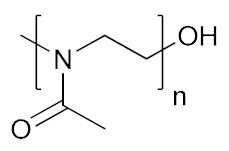	Hydrophilic, biocompatible	cell growth; tissue regeneration	[139,140]
Poly(vinyl alcohol)	PVA	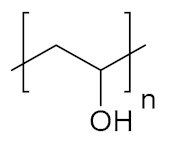	Hydrophilic, biocompatible	tissue engineering; regenerative medicine	[141,142,143]
Poly(acrylic acid)	PAAc	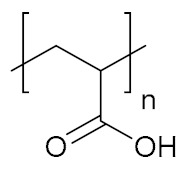	Hydrophilic, pH-responsive	cell growth	[144]
Poly [2-(dimethylamino)ethyl methacrylate]	PDMAEMA	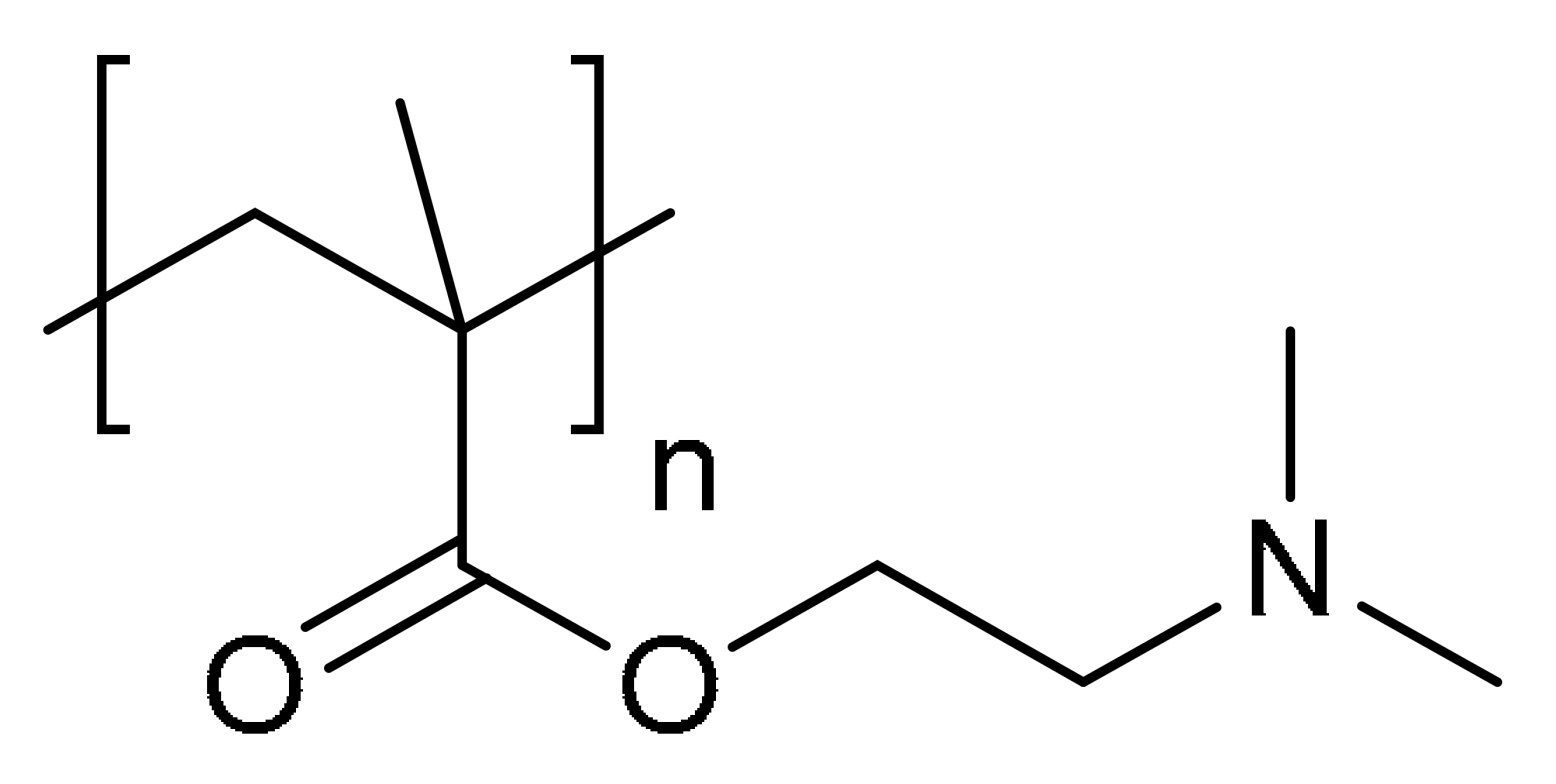	Hydrophilic, pH-responsive	biomolecule delivery, regenerative medicine	[145,146]
Poly(ethylenimine)	PEI	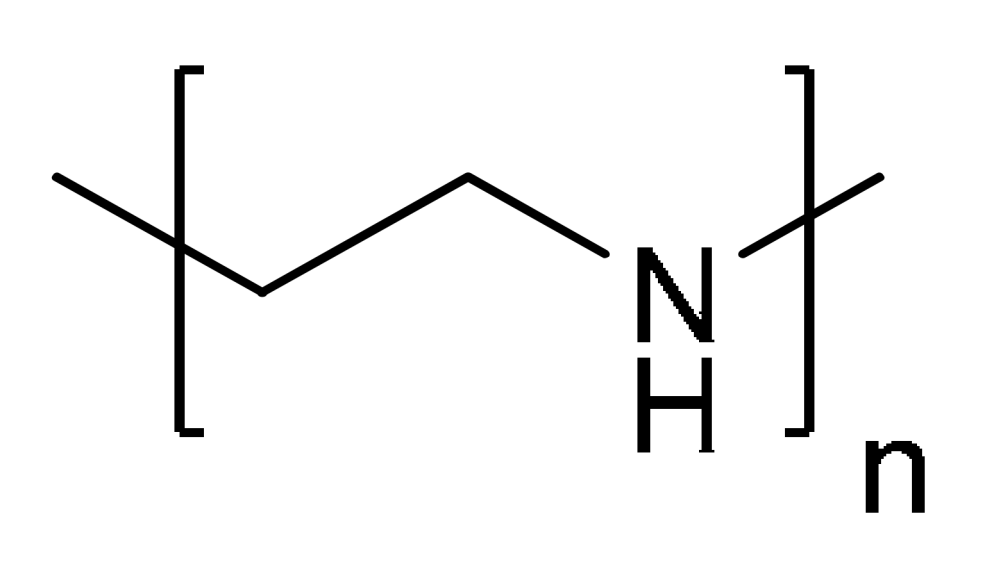	Hydrophilic, pH-responsive	biomolecule delivery	[147,148]
Poly(acrylamide)	PAAm	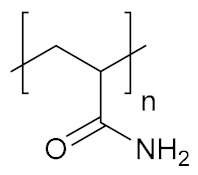	Hydrophilic, biocompatible	antigen sensing, regenerative medicine	[149]
Poly(N-vinyl pyrrolidone)	PNVP	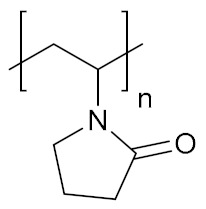	Hydrophilic, biocompatible	biomolecule delivery; tissue engineering	[150]
Poly(N-isopropyl acrylamide)	PNIPAM	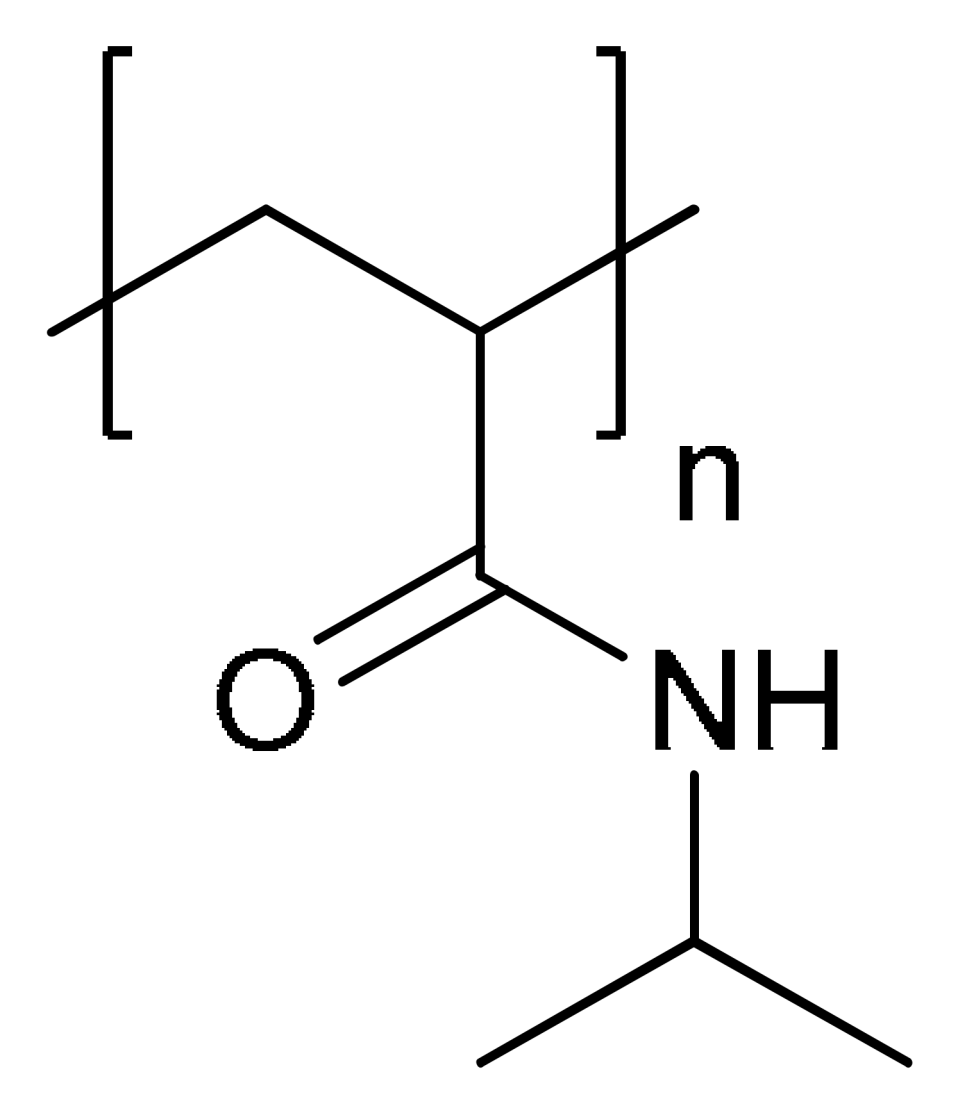	Hydrophilic, biocompatible, temperature responsive	biomolecule delivery; cell growth; tissue engineering	[145,151]
Poly(ε-caprolactone)	PCL	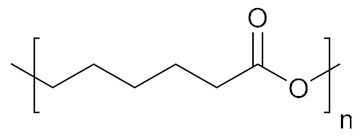	Hydrophobic, biodegradable	cell growth; tissue engineering	[152,153]
Poly(lactic acid)	PLA	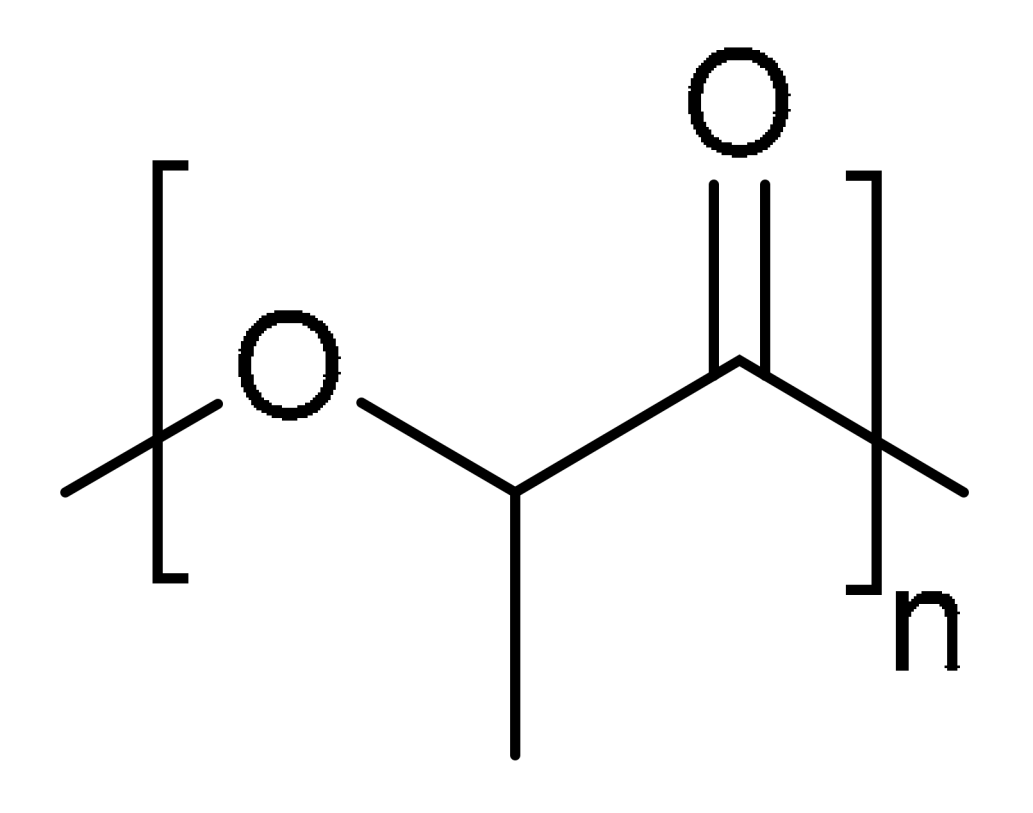	Hydrophobic, biodegradable, biocompatible	biomolecule delivery; tissue engineering	[154]
Poly(glycolic acid)	PGA	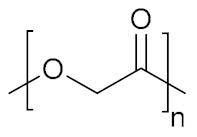	Slightly hydrophilic, biodegradable	Biomolecule delivery; tissue engineering	[155,156,157]
Poly(N-vinylcaprolactam)	PVCL	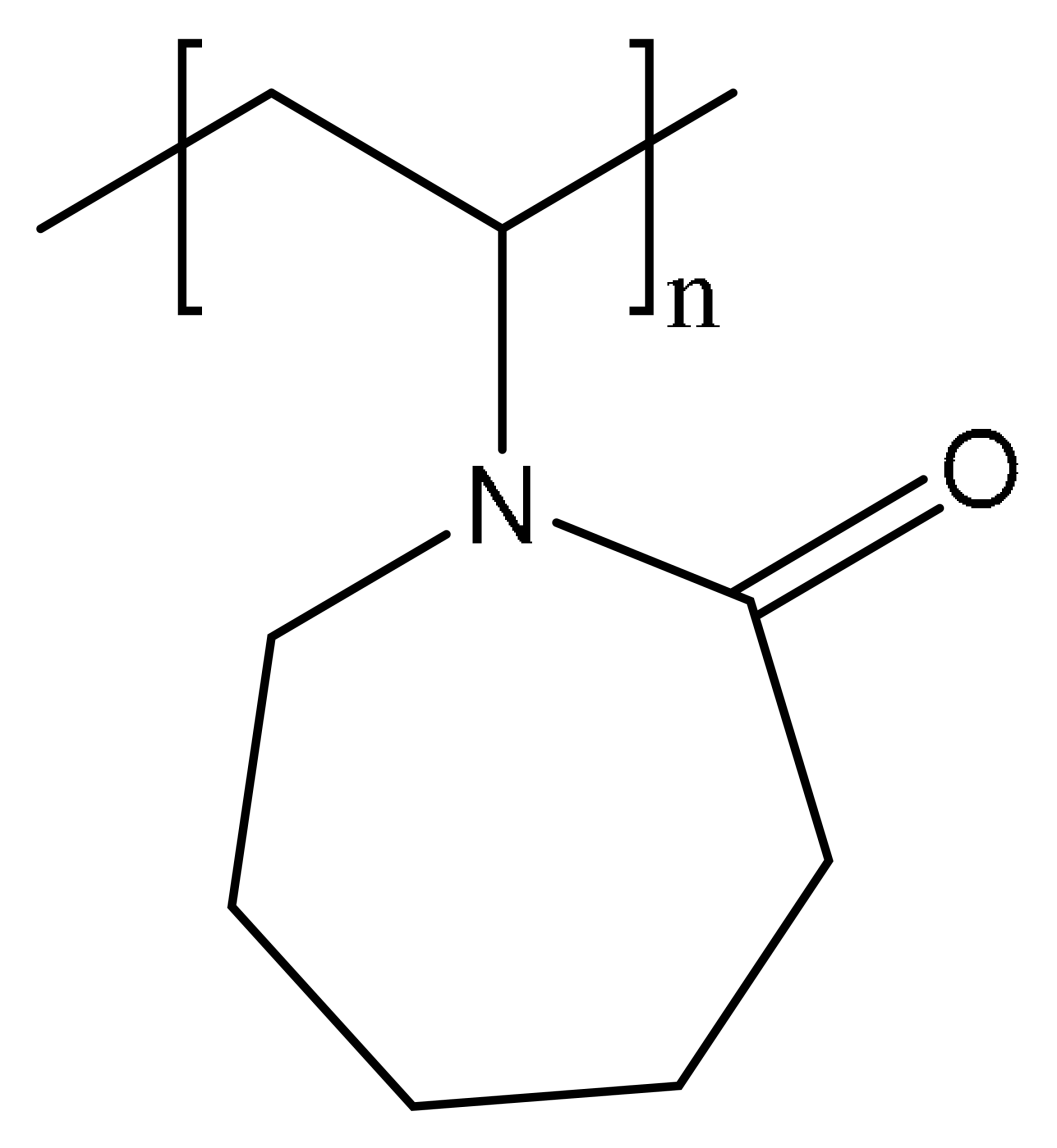	Hydrophobic, biocompatible, temperature responsive	Biomolecule delivery; theranostic; tissue engineering	[158,159,160]

**Table 2:** Summary of biomolecules entrapped within the matrix of hydrogels.

Biomolecule	Conjugation Methods	Applications	Refs.
Oligonucleotides	Covalent attachment, non-covalent crosslinking, electrostatic interactions	Drug delivery, cell culture	[171,172,173,174]
Proteins (GFs, enzymes)	Covalent attachment, non-covalent crosslinking (host-guest chemistry), electrostatic interactions	tissue regeneration	[175,176,177,178]
Carbohydrates	Covalent attachment, non-covalent attachment	tissue regeneration	[179,180]
Peptides	Covalent attachment	tissue adhesives, regeneration	[181,182,183,184]

## Abbreviations

**Abbreviation**	**Full Name**
Acs	Acetyl CoA Synthease
ATRP	Atom Transfer Radical Polymerization
°C	Degree Celsius
CGC	Critical Gelation Concentration
CL	Cross-linker
CMP	Collagen Mimetic Peptide
Cryo-SEM	Cryo-Scanning Electron Microscope
DAMA	*N*-(*N*′,*N*′-dicarboxymethyl aminopropyl) methacrylamide
DMSO	Dimethyl sulfoxide
DNA	Deoxyribonucleic acid
DTT	Dithiothreitol
EtOH	Ethanol
Fmoc	Fluorenylmethyloxycarbonyl chloride
*G’*	Storage Modulus
GF	Growth Factor
H_2_O	Water
hMSCs	Human Mesenchymal Stem Cells
HPMA	Hydroxypropyl methacrylate
IA	Itaconic acid
J	Joule
kPa	KiloPascal
LBM	Load Bearing Molecule
LCST	Lower Critical Solution Temperature
mAB	Monoclonal Anti Bodies
MADIX	Macromolecular Design by Interchange of Xantates
MeOH	Methanol
mm	Millimeter
NHS	N-Hydroxysuccinimide
nm	Nanometer
PAAc	Poly(acrylic acid)
PAAm	Poly(acryl amide)
PCL	Poly(ε-caprolactone)
PDMAEMA	Poly(2-(dimethylamino)ethyl methacrylate)
PHEMA	Poly(2-hydroxyethyl methacrylate)
PHPMA	Poly(2- hydroxypropyl methacrylate)
PEG	Poly(ethylene glycol)
PEI	Poly(ethylene imine)
PEODA	Poly(ethyleneoxide) diacrylate
PGA	Poly(glycolic acid)
PLA	Poly(lactic acid)
PLGA	Poly(lactic-glycolic acid)
PMOXA	Poly(2-methyl-2-oxazoline)
PNIPAM	Poly(*N*-ispropylacrylamide)
PNIPMAM	Poly(*N*-isopropylmethacrylamide)
PNVP	Poly(*N*-vinylpyrrolidone)
PPO	Poly(propylene oxide)
PU	Poly(urethane)
PVA	Poly(vinyl alcohol)
PVCL	Poly(vinyl caprolactam)
RAFT	Reversible Addition Fragmentation Chain Transfer
RGD	Arginylglycylaspartic acid
ROMP	Ring-Opening Metathesis Polymerization
T	Temperature
UCST	Upper Critical Solution Temperature
UV	Ultra Violet
VPTT	Volume Phase Transition Temperature
Wt%	Weight %
